# Investigating anabolic-androgenic steroid dependence and muscle dysmorphia with network analysis among male weightlifters

**DOI:** 10.1186/s12888-023-04781-1

**Published:** 2023-05-16

**Authors:** Morgan Scarth, Lars T. Westlye, Ingrid A. Havnes, Astrid Bjørnebekk

**Affiliations:** 1 Anabolic Androgenic Steroid Research Group, Section for Clinical Addiction Research, Division of Mental Health and Addiction, Oslo University Hospital, Postbox 4959, Nydalen, Oslo, 0424 Norway; 2 Department of Psychology, University of Oslo, Oslo, Norway; 3 Division of Mental Health and Addiction, Institute of Clinical Medicine, NORMENT, Oslo University Hospital, University of Oslo, Oslo, Norway; 4 KG Jebsen Centre for Neurodevelopmental Disorders, University of Oslo, Oslo, Norway; 5 Institute of Clinical Medicine, University of Oslo, Oslo, Norway

**Keywords:** Anabolic androgenic steroids, Network analysis, Muscle dysmorphia, Appearance and performance enhancing drugs

## Abstract

**Background:**

Anabolic-androgenic steroid (AAS) dependence has numerous adverse health consequences, and may be driven in part by body image concerns, primarily muscle dysmorphia. This study aims to further understand and identify potential clinical targets using network analyses of *AAS dependence* and *muscle dysmorphia* symptoms in males who used AAS and weightlifting controls.

**Methods:**

A sample of 153 men who currently or previously used AAS and 88 weight-lifting controls were recruited through social media and relevant online forums, and via posters and flyers distributed in select gyms in Oslo, Norway. Symptoms of AAS dependence and muscle dysmorphia were assessed using clinical interviews and standardized questionnaires. Severity of muscle dysmorphia symptoms were compared between the groups using independent samples t-tests. The following symptom networks were computed using Gaussian graphical modeling or mixed graphical modeling: (1) AAS dependence symptoms among men with AAS use (2) muscle dysmorphia symptoms among men with AAS use and weight-lifting controls in two separate networks, which were compared using a network comparison test, and (3) AAS dependence and muscle dysmorphia symptoms among men with AAS use.

**Results:**

In a network of AAS dependence symptoms, continuing use despite physical and mental side effects, using longer than planned, tolerance, and work/life interference were the most central symptoms. When comparing symptom structures of muscle dysmorphia between those who used AAS and controls, the most central symptoms in each group were exercise dependence and size/symmetry concerns, respectively. Men with AAS use demonstrated elevated muscle dysmorphia symptoms compared to controls, indicating that both the severity and structure of symptoms differ between these groups. In a network including both AAS dependence and muscle dysmorphia symptoms, no significant connections between symptom groups were identified.

**Conclusions:**

AAS dependence is complex, with correlated somatic and psychological challenges driving the symptom network, indicating that alleviating physical and mental health concerns during both AAS use and cessation is an important clinical target. Muscle dysmorphia symptoms related to taking action (diet, exercise, and supplement use) appear to cluster together more for those who use AAS than those who do not.

**Supplementary Information:**

The online version contains supplementary material available at 10.1186/s12888-023-04781-1.

## Background

Anabolic-androgenic steroids (AAS), comprising testosterone and synthetic derivatives, are typically used within the weight-lifting and bodybuilding communities to develop lean muscle mass. The global lifetime prevalence of AAS use is estimated to be 6.4% for men, and 1.6% for women, though these estimates are higher among certain populations including those with substance use disorder and athletes [1–3].

AAS use has been associated with a wide range of psychiatric and somatic health consequences including cognitive deficits, aggression, cardiovascular problems, and hypogonadism [4–11]. Approximately one-third of people who use AAS develop *dependence* [12, 13], which is characterized by AAS withdrawal, including hypogonadism symptoms (depression, anxiety, fatigue and sexual dysfunction), anxiety due to reduced muscle volume, and continued use despite negative impacts on physical and mental health [14–16]. AAS dependence is likely driven by both psychosocial and biological factors, including perceived positive effects such as increased strength and libido, decreased executive function, and a need to continue administering exogenous testosterone as natural production is disrupted [8, 17–19]. People with AAS dependence are at a greater risk for adverse health outcomes due to longer durations of use and higher doses. Thus, understanding the symptoms of and risk factors for AAS dependence is critical for providing optimal treatment to this population, and preventing severe and enduring side effects.

One of the proposed etiological factors for AAS dependence includes body image disorders, primarily *muscle dysmorphia* (MD) [16]. MD is a type of body dysmorphic disorder characterized by a persistent belief that one is too small or lacking musculature, despite having a normal or unusually muscular appearance [20, 21]. While certain behaviors and attitudes towards diet, exercise, and supplement use are not typically considered to be indicative of any pathology, these behaviors can be taken to extremes which influence functioning and quality of life [22]. MD is associated with appearance and performance-enhancing drug (APED) use, including AAS [23–26]. Strength athletes exhibit higher levels of MD symptoms compared to the general population, with a particularly high prevalence among bodybuilders, relative to other resistance-training practitioners [27–29]. Within this population, there are also significant associations between symptoms of MD and other risky health behaviors including eating disorders and compulsive exercise [29, 30]. In addition, MD symptoms are associated with other psychiatric problems including anxiety, neuroticism, depression and perfectionism, and thus may be a marker of greater mental health problems [27].

Previous studies indicate that MD is associated with initiating AAS use and duration of use, but the relationship to AAS dependence is less clear [31, 32]. Some findings suggest that AAS use is a perpetuating factor in the progression of MD [33], while others conclude that prolonged AAS use does not exacerbate symptoms of MD. However, specific symptoms, including social physique anxiety, or distress connected to the perceived evaluation of one’s body, may be related to severity of AAS dependence [34]. Thus, the relationship between MD and AAS dependence is not well understood, and further investigation at the symptom level may provide critical insights. Pharmacological use is common among individuals with MD, and includes the use of AAS, laxatives and diuretics. Previous studies among recreational exercisers indicate that MD symptoms including exercise dependence, drive for size/symmetry, and pharmacological use are associated with intentions to use APEDs, however the relationships among these symptoms and AAS dependence symptoms has not been investigated [35].

Understanding symptom structures of MD and AAS dependence, and the potential connections between these two groups of symptoms, may provide clinically relevant information about the development and prognosis of AAS dependence. Using network analysis, mental disorders and other complex conditions are understood as systems of interacting behaviors and experiences that dynamically influence one another, rather than manifestations of a specific latent pathology [36–38]. Undirected network analysis aims to identify the structure and strength of interactions between measured symptoms, without establishing directionality or causality. In these networks, nodes represent individual symptoms, and the strength of their connections, termed edges, reflect the associations between each symptom pair (often operationalized as a correlation). Principles from graph theory can be used to describe both global and local characteristics of the network, including node centrality and various estimates of overall node clustering, i.e. the degree to which the various symptoms represent sub-clusters or modularity. Network analysis has been applied to a variety of psychiatric disorders including eating disorders, PTSD, depression and substance use disorders (SUD), with potential clinical implications. For example, nodes which are central to the network can be interpreted as primary targets for clinical intervention, as improvement on these symptoms may contribute to alleviation of other symptoms in the network [37, 39, 40]. The persistence of the disorder or syndrome is thus determined by the extent to which the symptoms are interconnected. Nodes which connect two clusters within a network are deemed “bridge” nodes, which may indicate the pathway by which two clinical domains or disorders are connected [41], and may provide valuable information about mechanisms of comorbidities. For example, it was demonstrated that perfectionism symptoms bridge obsessive compulsive and eating disorders, suggesting that interventions targeting perfectionism behaviors may alleviate symptoms of both obsessive compulsive and eating disorders [42].

Previous studies have constructed dependence symptom networks for psychoactive substances, though this method has not been applied to APEDs. Network modeling supported the intuitive notion that using a drug more than planned, and spending a significant amount of time obtaining, using, or recovering from substance use were the most central symptoms of SUD [43, 44]. Furthermore, a network analysis of body dysmorphic disorder in a clinical sample found that interference in functioning because of appearance-related compulsions was the most central symptom [45]. These studies provide evidence that network analyses contribute to clinically relevant findings, and applying these methods to AAS dependence will similarly elucidate the most critical symptoms. Network analysis can identify differences in symptom structures between groups, including variations in MD symptom centrality between those who use AAS and those who do not, which may provide clinically relevant information about specific symptoms connections within those that use AAS. In addition, these methods can further understanding of the relationships among symptoms of AAS dependence and MD, potentially identifying key symptoms or clusters that drive these frequently co-occurring disorders. While there is a need for research in clinical MD samples, the present study examines behaviors and attitudes as symptoms rather than diagnoses to provide insight into the structure of these symptoms among males who regularly weight train, and those that use AAS [46].

With the goal of illuminating clinical targets for those seeking to cease AAS use, the current study aimed to identify the most central symptoms of AAS dependence and their connections to symptoms of MD using network analyses. Based on previous findings and current models of comorbidity we pursued the following hypotheses: (1) In a network of AAS dependence symptoms, using more or longer than planned will be most central. (2) In a comparison of MD symptom networks in AAS consumers and weight-lifting controls, supplement and pharmacological use will be more central to the AAS consumers’ network.

3) In a network of AAS dependence and MD symptoms among AAS consumers, we expect that the symptom “using more or for longer than planned” will be a bridge with MD symptoms.

## Methods

### Participants

The total sample consisted of 153 males with current or previous AAS use and 88 weight-lifting controls (WLC) participating in heavy resistance training, comprising exercise routines using heavy weights with the intention to increase strength and size of muscles. Participants were recruited through social media and online forums relevant to weight training, bodybuilding and AAS use, and via posters and flyers distributed in select gyms in Oslo, Norway and surrounding areas. Inclusion criteria were at least 18 years of age and for the WLC group one repetition bench press of at least 100 kg was required (220 pounds), with the aim of identifying controls with comparable lifestyle and training regimes to those in the AAS group. Current or previous AAS use for at least one year of cumulative use was required for inclusion in the AAS group. Controls were included if they reported no previous use of AAS or other APEDs listed on the World Anti-Doping Agency (WADA) list of Prohibited Substances, which was confirmed with a negative urine sample test as described in detail previously [47]. The current study is part of a longitudinal investigation of the effects of high-dose AAS use on brain health, and the sample is partly overlapping with those in previous publications [47]. Demographic information was self-reported including lifetime participation in specific training activities. Sample size was not calculated a priori. Participants were compensated with 1000 Norwegian kroner (~ 100 USD) at the first time point, and the cost of travel was compensated at follow-up (500 Norwegian kroner, ~ 50 USD).

### Measures

#### AAS dependence

AAS dependence was assessed by interview with trained study personnel using the Structured Clinical Interview for DSM-IV Axis II Disorders (SCID-II) for substance dependence, with adaptations for AAS by experts in the field, which has been found to have sufficient reliability and validity [13, 15, 48]. Participants were interviewed regarding the following seven symptoms of AAS dependence in their lifetime: tolerance, withdrawal, taking AAS in larger amounts or over a longer period than originally intended, a persistent desire or unsuccessful efforts to cut down or control use, a great deal of time spent obtaining, researching, using, or recovering from AAS in addition to training and preparing specific food, giving up social, occupational, or recreational activities due to AAS use, and continuing use despite persistent physical or psychological problems related to AAS use. Interviewers rated the symptoms on a scale from 1 to 3 (absent, subthreshold, present).

#### Muscle dysmorphia

The Muscle Dysmorphia Inventory (MDI) includes 27 items which aim to identify pathological attitudes and behaviors. The items are rated on a 6-point Likert scale (1 = never, 6 = always). The items are summed in six subscales: diet, evaluating how nutritional intake is regulated to achieve a muscular physique, supplement, evaluating the use of supplements to aid workouts and recovery, physique concealment, evaluating measures taken to hide one’s body or physique, exercise dependence, evaluating strict adherence to one’s workout regimen, size/symmetry, evaluating preoccupation with appearing muscular and fear of appearing small, and pharmacology, evaluating the use of steroids, laxatives, and diuretics [49].

### Ethical considerations

The study received approval from the Regional Committees for Medical and Health Research Ethics South East Norway (2013/601) and the data protection officer at Oslo University Hospital (18/09507) and was conducted in accordance with the Declaration of Helsinki.

### Data analysis

All analyses were completed using R version 4.1.1 [50]. AAS consumers were compared to WLC on all MDI subscales using independent t-tests, after assessment for normality. Each network was computed using complete case analysis with the available complete participant data from each interview and questionnaire (dependence network: *n = 139 AAS*, MD networks: *n = 88 WLC, 119 AAS*, combined dependence and MD network: *n = 105 AAS*). Multiple imputation was not carried out since missing data in this case implies that the relevant questionnaire/interview was completely missing, rather than missing items.

The first network was identified using the seven SCID items to evaluate relationships among dependence symptoms in AAS consumers by computing polychoric correlations between all items. Due to the ordinal nature of these scales, Gaussian Graphical Modeling (GGM) was used, where the edges of the network represent the regularized partial correlations between two symptoms, accounting for all other variables in the network. Model selection was based on the Extended Bayesian Information Criteria (EBIC). To reduce the risk of false-positive results, regularized estimation with graphical least absolute shrinkage and selection operator (LASSO) was used. The LASSO shrinks all edge-weights towards zero, leading to a sparse network structure. The degree of the penalty is controlled by the gamma hyperparameter, which was set to 0.50. Node centrality was calculated using the qgraph package [51] for expected influence (EI), which represents the summed edge weights of one node to other nodes in the network. EI has been reported to be a more reliable measure of centrality than closeness and betweenness, and takes the sign of correlation into account, unlike node strength [52–54].

To evaluate stability of the centrality measures, a case bootstrap with 2500 samples was used and centrality stability (CS) coefficient was calculated. The CS coefficient represents the maximum proportion of cases that can be dropped while maintaining 95% probability that the correlation between the node EI and edges of the original network and the bootstrapped network is at least 0.7. The CS coefficient should be at least 0.25, preferably above 0.50 [55]. To evaluate differences between edges and node EI within the network, a nonparametric bootstrap with 2500 samples, and the bootstrapped difference-test in the R package *bootnet* were used [56].

In the second stage of analysis, networks were computed for both the AAS and WLC groups using muscle dysmorphia symptoms, and networks were compared between the two groups. A similar GGM procedure as described above was used, but treating the MDI subscales as continuous variables and applying a nonparanormal transformation to account for the difference in scale using the R package *huge* [57]. A network comparison test was conducted using the R package *NetworkComparisonTest*, a two-tailed permutation method to evaluate differences between networks based on network structure invariance, global strength invariance, and edge invariance, with corresponding p-values [58]. In this study, 5000 iterations were used.

To investigate the connections between symptoms of AAS dependence and muscle dysmorphia, a network was constructed with SCID items (ordinal) and MDI items (continuous) using a mixed graphical model (MGM), where edges represent regression weights based on standardized data, to account for the differences in scale. Similar to the partial correlation network, regularized estimation with EBIC model selection with a gamma hyperparameter of 0.50 was used. The calculation of centrality measures and bootstrapping procedure was the same as for the partial correlation network, with the addition of bridge expected influence. This measure sums the edge weights of a node to nodes in another community (i.e. the sum of edge weights from a dependence symptom to muscle dysmorphia symptoms).

## Results

**Fig. 1:**
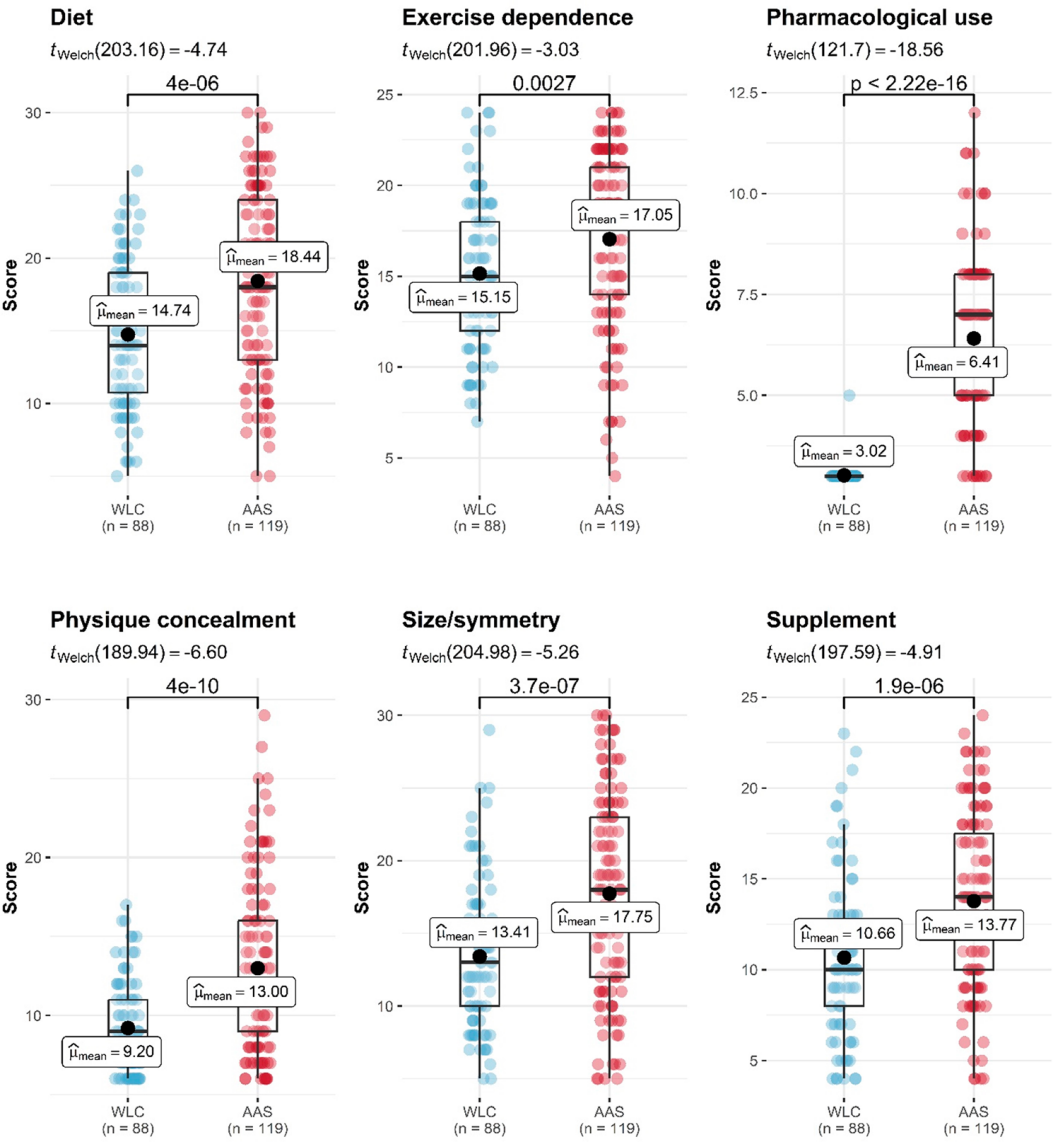
Distribution of Muscle Dysmorphia Inventory scale in weight-lifting controls (WLC) and anabolic androgenic steroid (AAS) consumers. N = 34 missing

Demographic and clinical information are reported in Table 1. The groups did not differ in age or height, however men in the AAS group reported fewer years of education and higher body mass index than WLC. The mean age for AAS initiation was 22.4 years (SD = 7.43) and the mean duration of use was 10.51 years (SD = 7.39). Bodybuilding was more prevalent among the AAS group (n = 58, 42.6%) than WLC (n = 12, 13.8%, *p* < .001). AAS consumers reported more MD pathology than non-using men, with statistically significant group differences on all MDI measures (Fig. 1). The mean time between the AAS dependence interview and MD questionnaire was 7.4 days (SD = 11.2).

**Table 1:** Demographic characteristics of the study population, characteristics of AAS use among users, and comparison of MD symptoms between users and weight-lifting controls

	WLC	AAS	*p*
n	88	153	
Age (mean (SD))	33.29 (10.14)^a^	35.78 (9.95)^b^	0.069
Education (years)	16.12 (2.60)	14.27 (2.38)^c^	< 0.001
Weight (kg)	90.78 (12.99)	99.10 (15.44)^c^	< 0.001
Height (cm)	181.49 (6.75)	181.55 (7.02)^c^	0.945
BMI (kg/m^2^)	27.55 (3.61)	30.03 (4.14)^c^	< 0.001
**Type of training (lifetime) (n(%))** ^**d,e**^			
Bodybuilding	12 (13.8)	58 (42.6)	< 0.001
Strongman	2 (2.3)	11 (8.1)	0.132
Combat sports	11 (12.6)	30 (22.1)	0.111
Weightlifting	13 (14.9)	17 (12.5)	0.749
Recreational exercise	33 (37.9)	43 (31.6)	0.409
Other sports	71 (81.6)	73 (53.7)	< 0.001
Debut age AAS	-	22.40 (7.43)^c^	
Duration of AAS use (years)	-	10.51 (7.39)^c^	
**SCID** ^f^			
Tolerance (n(%))			
*Absent*		28 (20.1)	
*Subthreshold*		46 (33.1)	
*Present*		65 (46.8)	
Withdrawal			
*Absent*		26 (18.7)	
*Subthreshold*		43 (30.9)	
*Present*		70 (50.4)	
Use longer than planned			
*Absent*		27 (19.4)	
*Subthreshold*		38 (27.3)	
*Present*		74 (53.2)	
Unable to stop			
*Absent*		52 (37.4)	
*Subthreshold*		45 (32.4)	
*Present*		42 (30.2)	
Time spent			
*Absent*		36 (25.9)	
*Subthreshold*		45 (32.4)	
*Present*		58 (41.7)	
Interferes with work/life			
*Absent*		64 (46.0)	
*Subthreshold*		40 (28.8)	
*Present*		35 (25.2)	
Physical/mental problems			
*Absent*		27 (19.4)	
*Subthreshold*		51 (36.7)	
*Present*		61 (43.9)	

*WLC = weight-lifting controls, AAS = anabolic-androgenic steroids, SCID = Structured Clinical Interview for DSM-IV, MDI = Muscle Dysmorphia Index. The following superscripts correspond to n missing: a = 1, b = 11, c = 15, d = 1 (WLC group), e = 17 (AAS group), f = 14*

### Dependence network

**Fig. 2:**
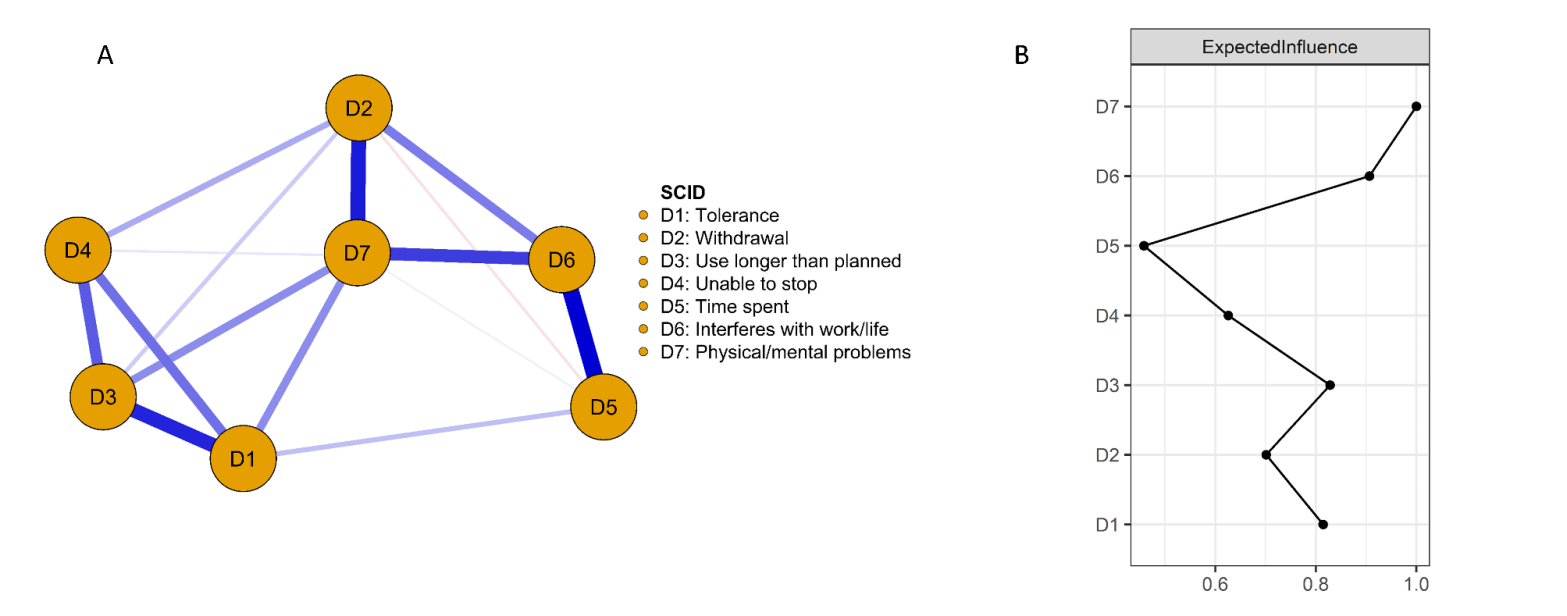
Network of AAS dependence symptoms among men who use AAS **(A)** and standardized expected influence **(B)**. In panel A, blue lines represent regularized partial correlations, where thicker lines represent stronger correlations. Red lines represent negative regularized partial correlations. In panel B, symptoms further to the right indicate greater expected influence

Figure 2 illustrates the network of SCID symptoms (nodes) among AAS consumers connected by edges differing in weight (regularized partial correlations), with the thickness of the line corresponding to the strength of the bidirectional association. The strongest connection in this network was between *time spent* (obtaining and using AAS in addition to time spent on related lifestyle factors) and *interferes with work/life*.

Based on expected influence (EI), *continuing use despite physical/mental problems* was the most central symptom, but the EI did not statistically significantly differ from *interferes with work/life*, *use longer than planned*, *withdrawal*, or *tolerance*. *Continuing use despite physical/mental problems* was most strongly associated with *withdrawal* and *interferes with work and personal life*. *Time spent* was the least central symptom. The CS coefficients, which represent the maximum drop proportions to retain correlation of 0.7 in at least 95% of the samples, indicated that the stability of both EI (0.36) and edge weights (0.44), were acceptable [55]. See supplemental materials for results of bootstrapped edge weight difference, EI differences, and stability.

### MD networks and comparison

**Fig. 3:**
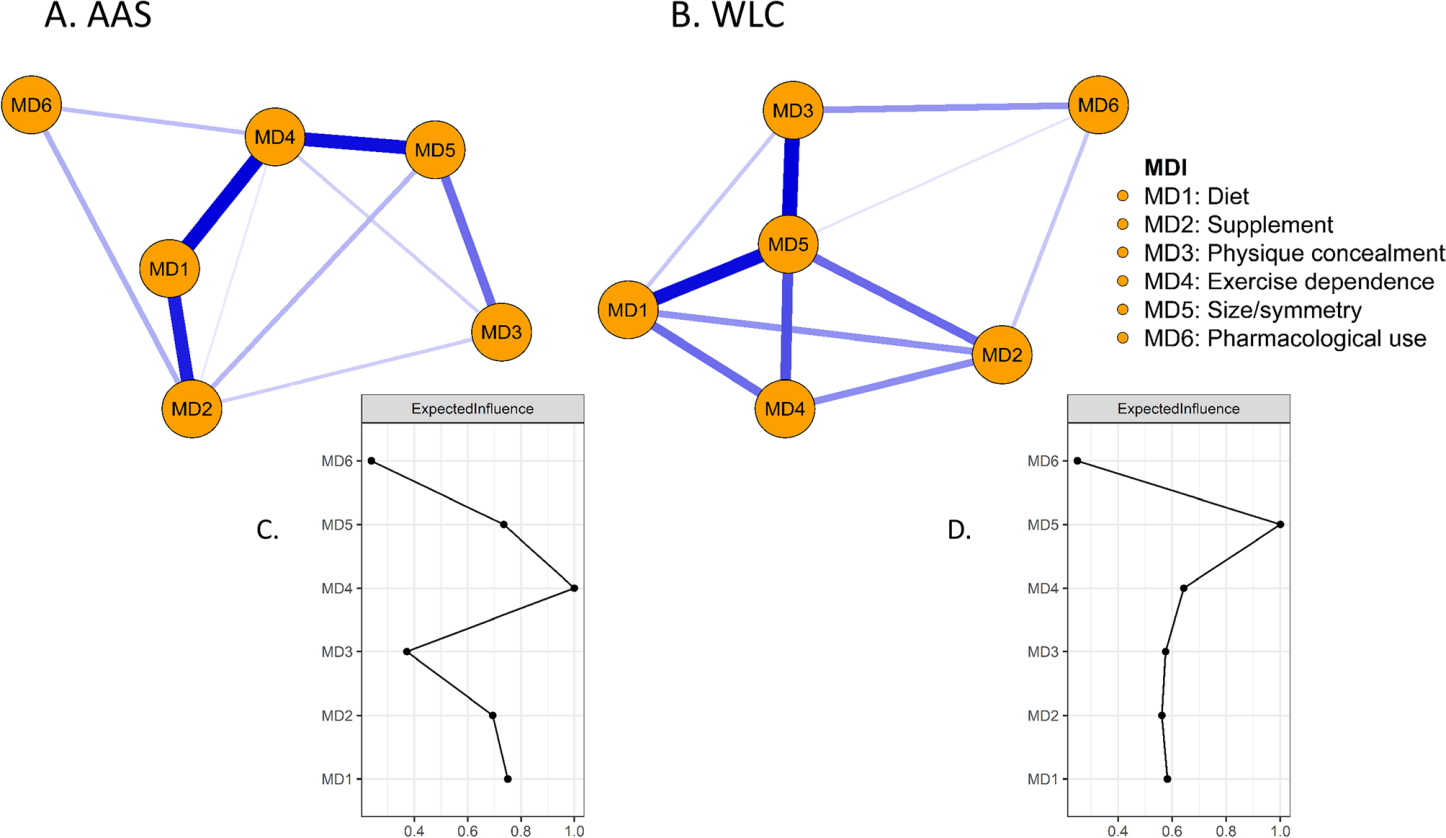
Networks of MDI scales in AAS consumers (panel **A**) and WLC (panel **B**) and standardized expected influence of each node in AAS consumers (panel **C**) and WLC (panel **D**). In panels A and B, blue lines represent positive regularized partial correlations. In panels C and D, symptoms further to the right indicate greater expected influence

**Fig. 4:**
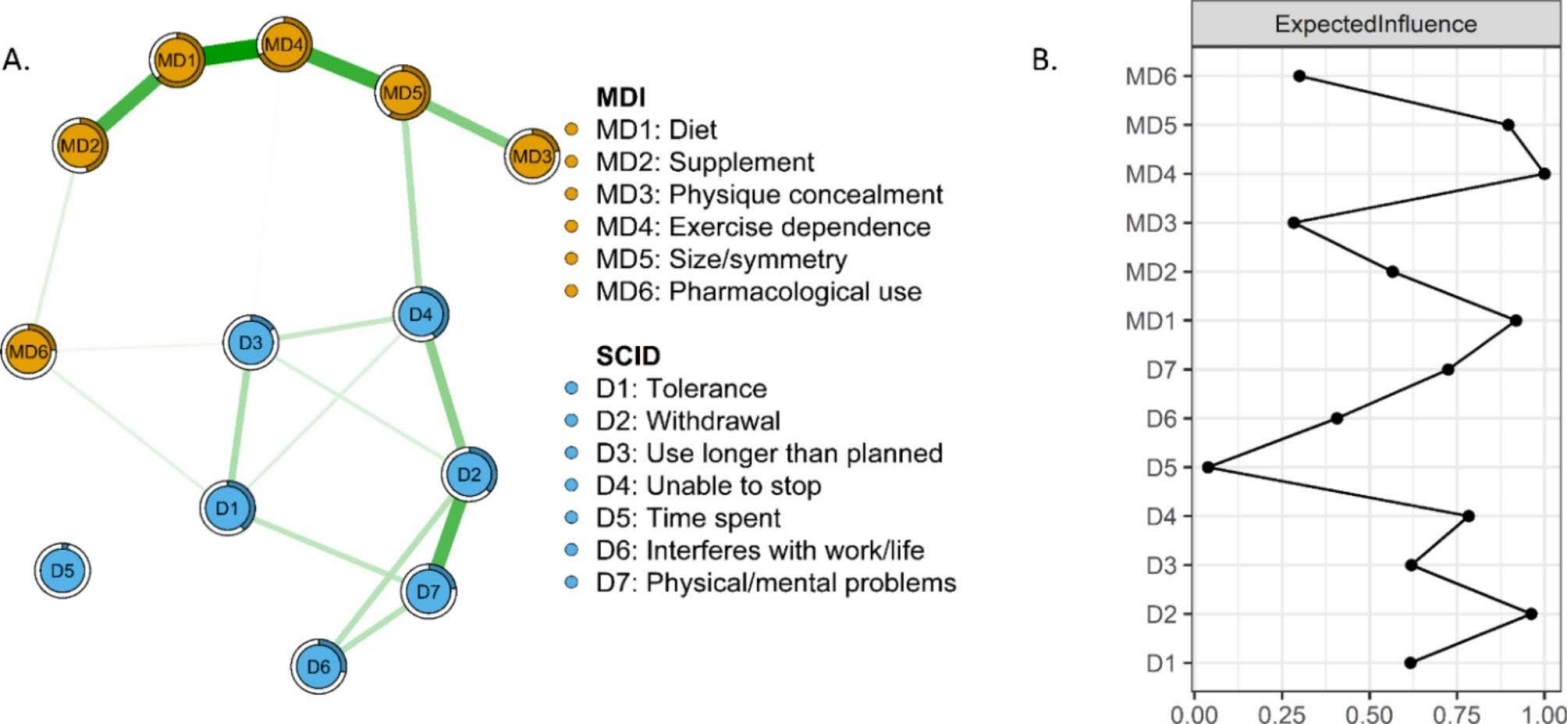
Mixed graphical modeling network of AAS dependence and muscle dysmorphia symptoms among AAS consumers (panel **A**) and standardized expected influence of symptom (panel **B**). In panel A, green lines represent positive relationships. The rings around nodes indicate variance of a given variable with the shaded part representing that proportion of the variance that can be explained by the connected nodes. In panel B, symptoms further to the right indicate greater expected influence

Figure 3 illustrates the network of MDI subscales in AAS consumers. *Exercise dependence* demonstrated the greatest EI, with strong connections to *diet* and *size/symmetry*. This network demonstrated acceptable stability (CS-EI = 0.597, CS-edge = 0.597). Figure 3B illustrates the MD network for WLC, where *size/symmetry* was most central, and correlated strongly with *physique concealment* and *diet.* This network demonstrated below acceptable stability (CS-EI = 0.05, CS-edge = 0.05). Bootstrap test results for the networks presented in Figs. 3 and 4 can be found in the supplemental materials.

The results of the network comparison test indicated no statistically significant difference in network invariance, representing the maximum difference in edge weights of the observed networks (*p* = .16). There was no statistically significant difference in global strength, representing the sum of all edge weights (*p* = .20). Two edges were significantly different between the groups, where the association between *diet* and *exercise dependence* was stronger among AAS consumers (*p* = .04), and the association between *diet* and *size/symmetry* was stronger among the WLC group (*p* < .01). Based on EI, *exercise dependence* was more central among the AAS group (*p =* .01). Full results of the NCT can be found in the supplemental material.

### Mixed graphical model of dependence and MD symptoms in AAS consumers

Figure 4 shows the network across dependence and MD symptoms, suggesting potential small associations between SCID and MDI items, however the network does not appear stable and thus findings must be interpreted with caution. *Exercise dependence*, *diet*, and *withdrawal* had the highest EI, however, the differences between the EI of these nodes and all others in the network were not statistically significant. The network suggests some connections between dependence and MD symptoms. Specifically, *unable to stop* appears associated with *size/symmetry*, and *use longer* appears associated with *pharmacological use*. However, after the bootstrap analysis there were no statistically significant differences among the nodes on bridge EI (see supplemental materials). This network had acceptable stability for centrality and EI, but low stability for bridge EI (CS-EI = 0.51, CS-edge = 0.51, CS-bridge = 0). Full results of the bootstrapped tests can be found in the supplemental material.

## Discussion

In this network analysis of dependence symptoms in AAS consumers, *continuing use despite physical/mental problems* was the most central symptom, and thus a relevant clinical target. However, *life interference*, *tolerance*, and *using longer than planned* were also highly influential, indicating the connections and importance of both physical and mental symptoms. Our findings also demonstrated higher severity of symptoms of MD in AAS consumers compared to WLC. Additionally, the structures of these symptoms appear to vary between the two groups, suggesting that experiences of MD symptoms differ between those who use AAS and those who do not.

The dependence network identified in this study differs from previous networks based on SUD patients using psychoactive substances [43, 44]. For example, in the present study, *time spent* had the least influence on a network of AAS dependence symptoms. Conversely, in previous studies this symptom was the most central in a network of dependence and harmful substance use symptoms among SUD patients [44]. In the same study, *continuing use despite physical/mental problems*, had high strength in the networks of several substances (alcohol, cocaine, opioids, and stimulants), but had relatively low strength in the cannabis network, suggesting that the symptom structure of AAS dependence may be more similar to stimulants or opioids than cannabis [44]. It is possible that time spent on substance use is less influential in the AAS network due to the difference in lifestyle and motivation for use of AAS compared to certain psychoactive substances. People who use AAS and other APEDs often report strict diet and exercise routines, requiring significant amounts of time, in addition to forming friendships around these objectives, which may normalize these regiments [59, 60]. Furthermore, people who use AAS often report spending a considerable amount of time researching these compounds before commencing use [61], and likely spend considerable amounts of time on other aspects of bodybuilding or fitness including training, diet and supplement use, indicating that further analyses are needed to evaluate the validity of this symptom in AAS dependence specifically.

An earlier investigation of dependence symptoms across all psychoactive substance classes identified *using more/for longer than planned* as the most central symptom [43]. Interestingly, in a subsequent network analysis of only those who used opioids, both *interference with work/life* and *using more/longer than planned* were identified as highly central symptoms, which demonstrates some similarity with the network identified in this study [43]. Although speculative, this similarity may be partially explained by shared reward mechanisms for opioid and AAS dependence involving opioid receptors in the dopamine system of the ventral tegmental area, which is often implicated in substance use and addiction [62–64]. In addition, common personality traits and cognitive dysfunctions have been identified in both AAS and opioid consumers [8, 65, 66]. Furthermore, some of the strong edges in the AAS dependence network may be explained by similarly worded or clearly related items. For example, spending significant amounts of time acquiring and using AAS may take away from time spent on work and personal life, and increasing doses may be representative of a tolerance to AAS. It is therefore logical that *time spent* is correlated with *work/life interference*, and *using more/longer than planned* is correlated with *tolerance* in the network.

The differences in MD symptoms between males who used AAS and WLC in the current study align with previous findings, which have identified elevated pathology among those using AAS and other APEDs [26, 67–69]. Furthermore, increased body image disturbance has previously been associated with increased risk of APED use and more time spent exercising weekly [70]. More broadly, body image concerns likely drive a range of practices, including eating disorders and exercise addiction that put individuals’ health at risk in pursuit of performance or aesthetic goals [26, 71, 72]. Many practices related to body dissatisfaction may co-occur, as previous research has identified associations between AAS use and eating disorder psychopathology [72]. In the present study, the structure of MD symptoms varied between AAS consumers and WLC, indicating that *exercise dependence* was the most central symptom for AAS consumers, whereas *size/symmetry* was most central in the WLC network, suggesting potential differences in body image concerns manifesting as stronger connections among behaviors rather than attitudes. Furthermore, people who use AAS regularly report rewarding effects, including enhanced mood and increased self-esteem while using AAS, which may decrease negative feelings regarding musculature or physique concealment while on-cycle [73]. While those who used AAS still reported higher levels of physique concealment, the positive effects while on-cycle may partially explain the differences in MD symptom structure. The group differences of *pharmacological use* suggest that in addition to abstaining from AAS, those in the WLC group use few or no laxatives or diuretics, whereas a significant portion of the AAS group also use laxatives and/or diuretics in addition to AAS, which is in line with earlier studies indicating that APED polypharmacy is common among AAS consumers [74]. In addition, the low inter-individual variation in pharmacological use among WLC may explain the low centrality of this symptom.

Furthermore, certain edges differed between the networks in the two groups, particularly those related to diet. Within the AAS group, diet appeared strongly related to both supplement use and exercise dependence, whereas in WLC diet was most strongly associated with size/symmetry concerns. This suggests that MD for AAS consumers consists of particularly strong connections among symptoms that involve taking action to change ones appearance (diets, supplements, training), whereas the WLC network demonstrates greater influence of nodes representative of internalized thoughts and feelings such as wanting to conceal their physiques. AAS consumers have previously demonstrated elevated levels of impulsivity and neuroticism, which may contribute to using more extreme practices and willingness to accept increased risk to achieve their desired results compared to those who do not use AAS [65, 75].

Motivation for AAS use may partially explain variation in MD symptoms among those who use AAS, as individuals reporting appearance concerns as their main motivation for use are more likely to demonstrate body image psychopathology than those driven by performance enhancement [76]. However, MD and body image concerns have been reported as both a cause for, and a result of, AAS use [73]. Thus, motivation for initiation of and continued AAS use should be taken into consideration in clinical settings when evaluating body image concerns. Moreover, in our sample, a greater proportion of AAS consumers participate in bodybuilding, which places a high value on appearance. A previous network analysis of body dysmorphic disorder patients identified interference in functioning due to appearance related compulsions as the most central symptom [45], which is more similar to the WLC network in the current study. However, it is important to note this study used a different tool to evaluate dysmorphia symptoms in a non-clinical sample, and that the WLC group in this study demonstrated fewer symptoms of dysmorphia on all scales compared to AAS consumers.

Contrary to our expectation, few connections were identified between MD and dependence symptoms. Small connections may exist between being *unable to stop* using AAS and *size/symmetry* concerns, however the results did not identify any statistically significant differences in bridge EI, though this study may lack power to detect bridges between these symptom groups. The findings indicate clear differences in MD symptoms between WLC and AAS consumers, but no clear connections between MD symptoms and AAS dependence symptoms. It is possible that these associations cannot be detected on the symptom level, but that group differences between people who use AAS with and without dependence may differ on a combined measure of MD. It may also be the case that MD is related to AAS use, but not dependence, in line with previous findings [31]. However, greater physique anxiety has been associated with more severe dependence, indicating some heterogeneity within the few studies on this relationship [34]. This may also reflect the heterogeneous nature of people with AAS dependence and MD symptoms, where intra-individual networks could reveal connections between the symptom groups within a single person.

### Limitations

The current study has some limitations. The sample size is relatively small for a network analysis, particularly for the MGM analysis. However, this is a hard-to-reach population and the current sample is derived from the largest study to date investigating the effects of AAS on brain health. These results should thus be interpreted with caution, and bootstrapped results and CS coefficients were computed to provide a view of how stable and reliable the results are. In addition, not all participants completed all questionnaires, which may bias results, particularly when comparing the results of the individual networks (of SCID and MDI networks) with the MGM network, as the samples are not identical. This study also relies on self-report measures, which may introduce bias, although the instruments used have been to found to have good validity. The AAS group comprised both past and previous use, which may influence the reporting of dependence symptoms in particular. The cross-sectional nature of this study does not allow for establishing the direction of the relationships among dependence and MD symptoms. Additionally, the MDI scale “pharmacological use” inquired about laxative, diuretic, and anabolic steroid use, the last of which will be homogenous within each of the groups, which may influence the outcome of these networks. Finally, the study sample does not represent a population meeting the clinical criteria for MD. However, the current study aimed to identify relationships among symptoms of AAS dependence and MD in a relevant demographic group, in order to provide an insight into the relationships among these symptoms for men engaged in heavy resistance training, though the results may not be generalizable outside of the Norwegian context.

## Conclusion

The current findings from a symptom network analysis support that *continuing use despite negative side effects* is a key symptom of AAS dependence. *Life interference*, *tolerance*, and *using longer than planned* had similar degrees of influence on the symptom network, suggesting that alleviating both psychiatric and physical symptoms will be imperative for those with dependence symptoms who want to cease AAS use. Thus, future studies should explore the heterogeneity of these effects, as people who use AAS may be more likely to seek treatment as a result of mental, rather than somatic, health concerns [77]. The problems related to ongoing AAS use include both physical and mental health issues such as gynecomastia, cardiovascular and liver pathology, mood swings, irritability, and aggressiveness [78, 79]. Withdrawal symptoms related to hypogonadism are commonly experienced for months during off periods [80], contributing to continued AAS use to relieve these symptoms. The most central symptoms to the network indicate that this cycle of experiencing side effects and potentially continuing AAS use to alleviate these side effects typifies AAS dependence. Hence, clinicians should aim to identify this experience, and be aware that reducing the discomfort experienced by those who want to quit AAS use is critical.

Additionally, the experience of MD symptomology may differ between those that use AAS and those that do not, in both severity and structure of symptoms. MD symptoms indicative of taking action (diet, exercise, and supplement use) appear to cluster together more for those who use AAS than those who do not, which may be a marker of use for clinicians. In clinical settings, motivation to initiate or continue AAS use should be addressed in order to provide personalized care. Considering that body dissatisfaction is associated with poorer mental health, and greater psychopathology [81, 82], clinicians, particularly mental health care providers, should take the use or planned use of AAS, and potentially other APEDs, into account when working with men experiencing MD symptoms or body dysmorphic disorders. Additionally, MD symptoms and body image should be evaluated when working with men who use AAS, and their psychological response to changes in muscle volume following a cycle or when attempting to cease use should be explored.

Further studies and larger sample sizes are required to verify the symptom structures identified in the current study, particularly the network including both AAS dependence and MD symptoms. Additionally, network analysis provides a clinically relevant approach to psychiatric disorders, which allows for embracing the complexity of these disorders, and should be applied to AAS dependence and muscle and body dysmorphic disorder more broadly in future research [83, 84].

## Electronic supplementary material

Below is the link to the electronic supplementary material.

Supplementary Material 1: Results of network analyses bootstrap tests

## Data Availability

The data supporting the findings of this study are available upon reasonable request. The data are not publicly available due to their sensitive nature, where our ethical approval prevents us from sharing data beyond named collaborators. Further inquiries can be directed to the corresponding author (MS).

## List of Abbreviations

AAS: Anabolic-androgenic steroids
APED: Appearance and performance-enhancing drugs
CS: Centrality stability
EI: Expected influence
MGM: Mixed graphical modeling
MD: Muscle dysmorphia
MDI: Muscle dysmorphia index
NCT: Network comparison test
SCID: Structured clinical interview
WLC: Weight-lifting control
