# Supplementary material for: Investigating anabolic-androgenic steroid dependence and muscle dysmorphia with network analysis among male weightlifters
Source: BMC Psychiatry. 2023 May 16;23:342. doi: 10.1186/s12888-023-04781-1 (PMC10186641; doi:10.1186/s12888-023-04781-1)
Supplement: Supplementary file 1 — Supplementary Material 1: Results of network analyses bootstrap tests [file 12888_2023_4781_MOESM1_ESM.docx]

Supplementary materials

[Dependence network 2](#_Toc126064836)

[Figure S1. Expected influence stability of AAS dependence network 2](#_Toc126064837)

[Figure S2. Edge weight bootstrap of AAS dependence network 3](#_Toc126064838)

[Figure S3. Bootstrap difference node expected influence of AAS dependence network 4](#_Toc126064839)

[Figure S4. Bootstrap difference edge weights of AAS dependence network 5](#_Toc126064840)

[MDI network: AAS users 6](#_Toc126064841)

[Figure S5. Expected influence stability of muscle dysmorphia network in AAS users 6](#_Toc126064842)

[Figure S6. Edge weight stability of muscle dysmorphia network in AAS users 7](#_Toc126064843)

[Figure S7. Bootstrap difference node expected influence of muscle dysmorphia network in AAS users 8](#_Toc126064844)

[Figure S8. Bootstrap difference edge weight of muscle dysmorphia network in AAS users 9](#_Toc126064845)

[MDI network: WLC 10](#_Toc126064846)

[Figure S9. Expected influence stability of muscle dysmorphia network in WLC 10](#_Toc126064847)

[Figure S10. Edge weight stability of muscle dysmorphia network in WLC 11](#_Toc126064848)

[Figure S11. Bootstrap difference expected influence of muscle dysmorphia network in WLC 12](#_Toc126064849)

[Figure S12. Bootstrap difference edge weights of muscle dysmorphia network in WLC 13](#_Toc126064850)

[Table S1. Network Comparison Test results 14](#_Toc126064851)

[Dependence + MDI network 15](#_Toc126064852)

[Figure S13. Expected influence stability of dependence and muscle dysmorphia network 15](#_Toc126064853)

[Figure S14. Edge weight stability of dependence and muscle dysmorphia network 16](#_Toc126064854)

[Figure S15. Bridge strength stability of dependence and muscle dysmorphia network 17](#_Toc126064855)

[Figure S16. Bootstrap difference expected influence of dependence and muscle dysmorphia network 18](#_Toc126064856)

[Figure S17. Bootstrap difference edge weights of dependence and muscle dysmorphia network 19](#_Toc126064857)

[Figure S18. Bootstrap difference bridge strength of dependence and muscle dysmorphia network 20](#_Toc126064858)

## Dependence network

### Figure S1. Expected influence stability of AAS dependence network


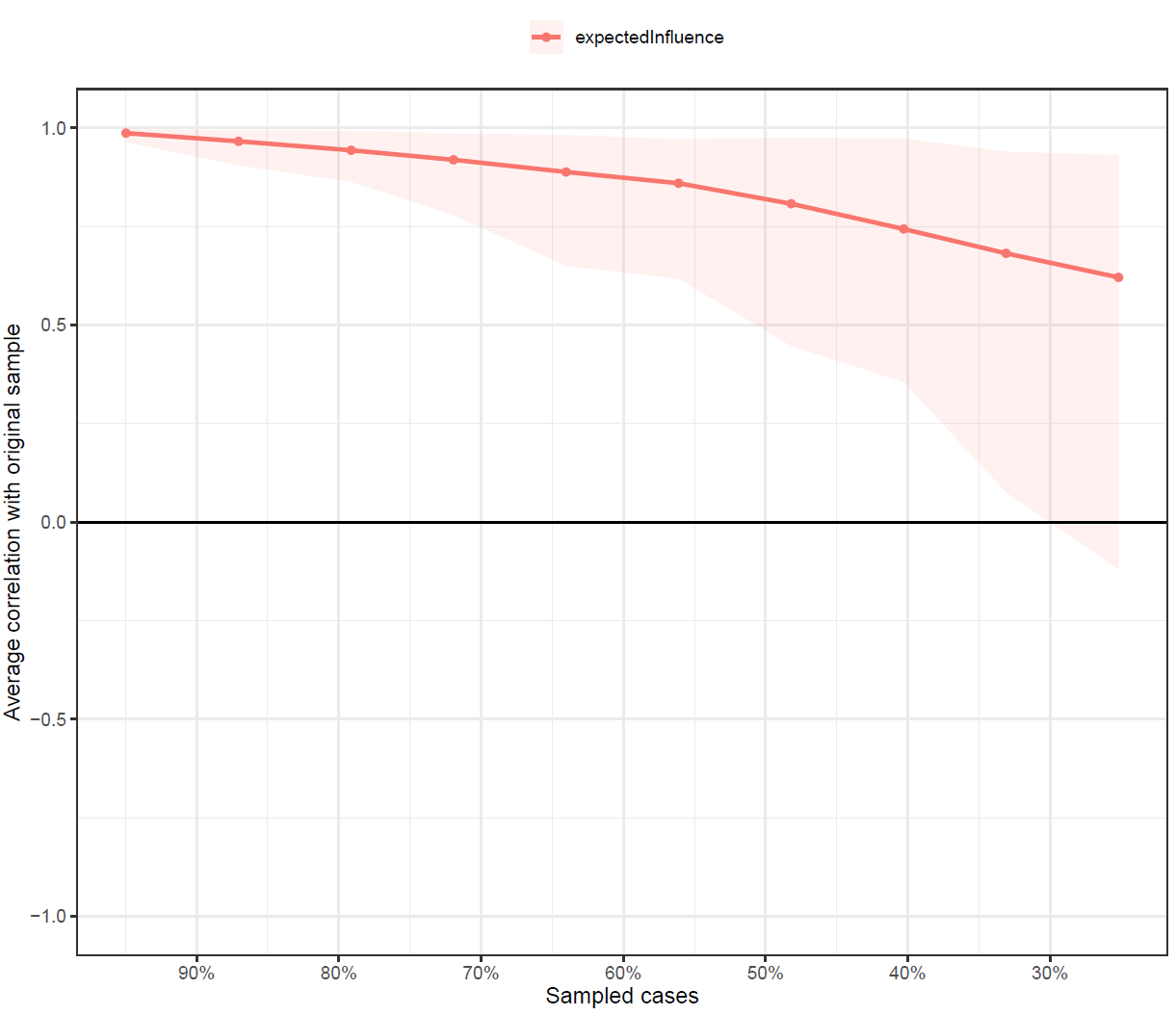


### Figure S2. Edge weight bootstrap of AAS dependence network


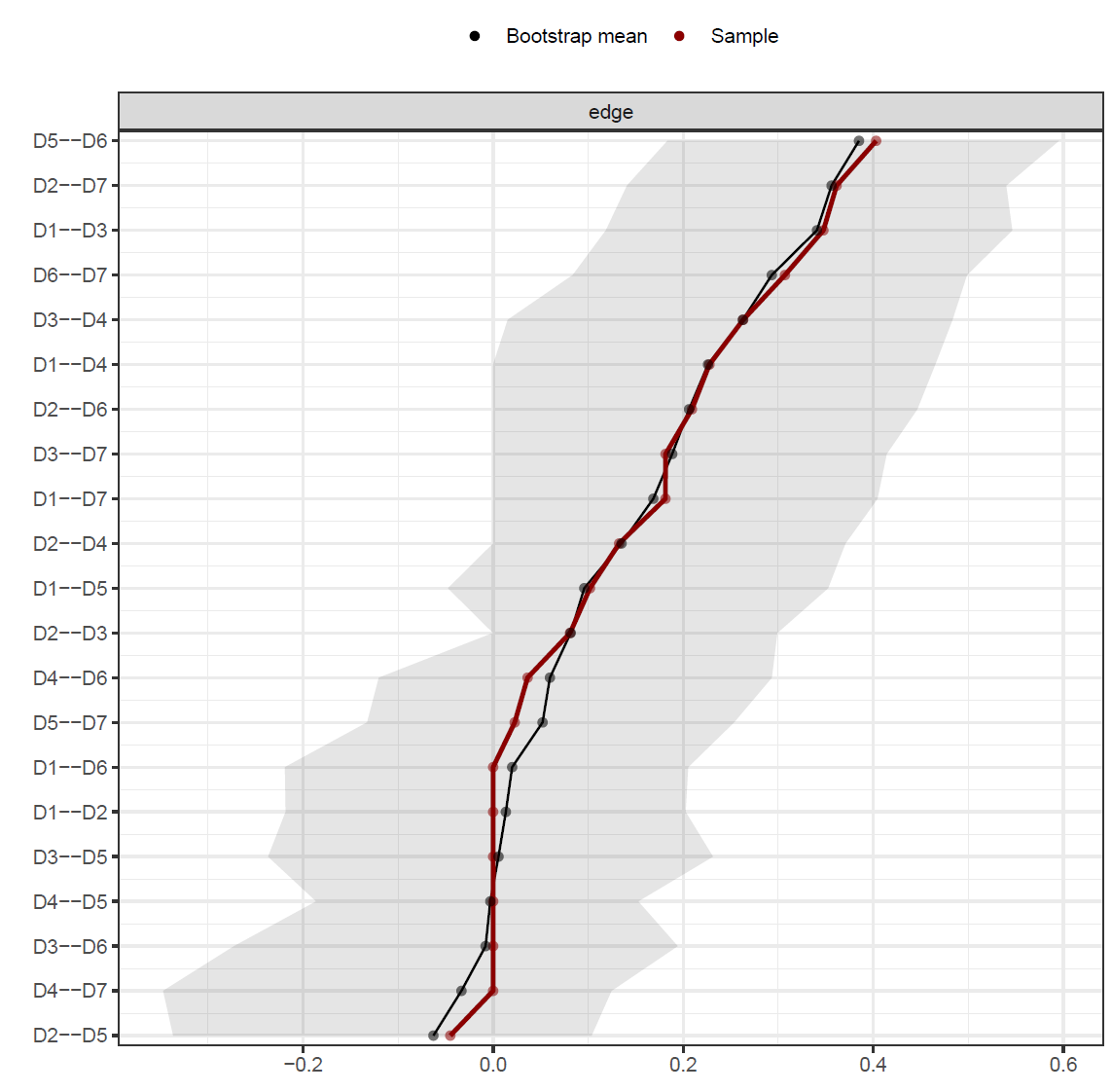


*D1: Tolerance, D2: Withdrawal, D3: Use longer than planned, D4: Unable to stop, D5: Time spent, D6: Interferes with work/life, D7: Physical/mental problems*

### Figure S3. Bootstrap difference node expected influence of AAS dependence network


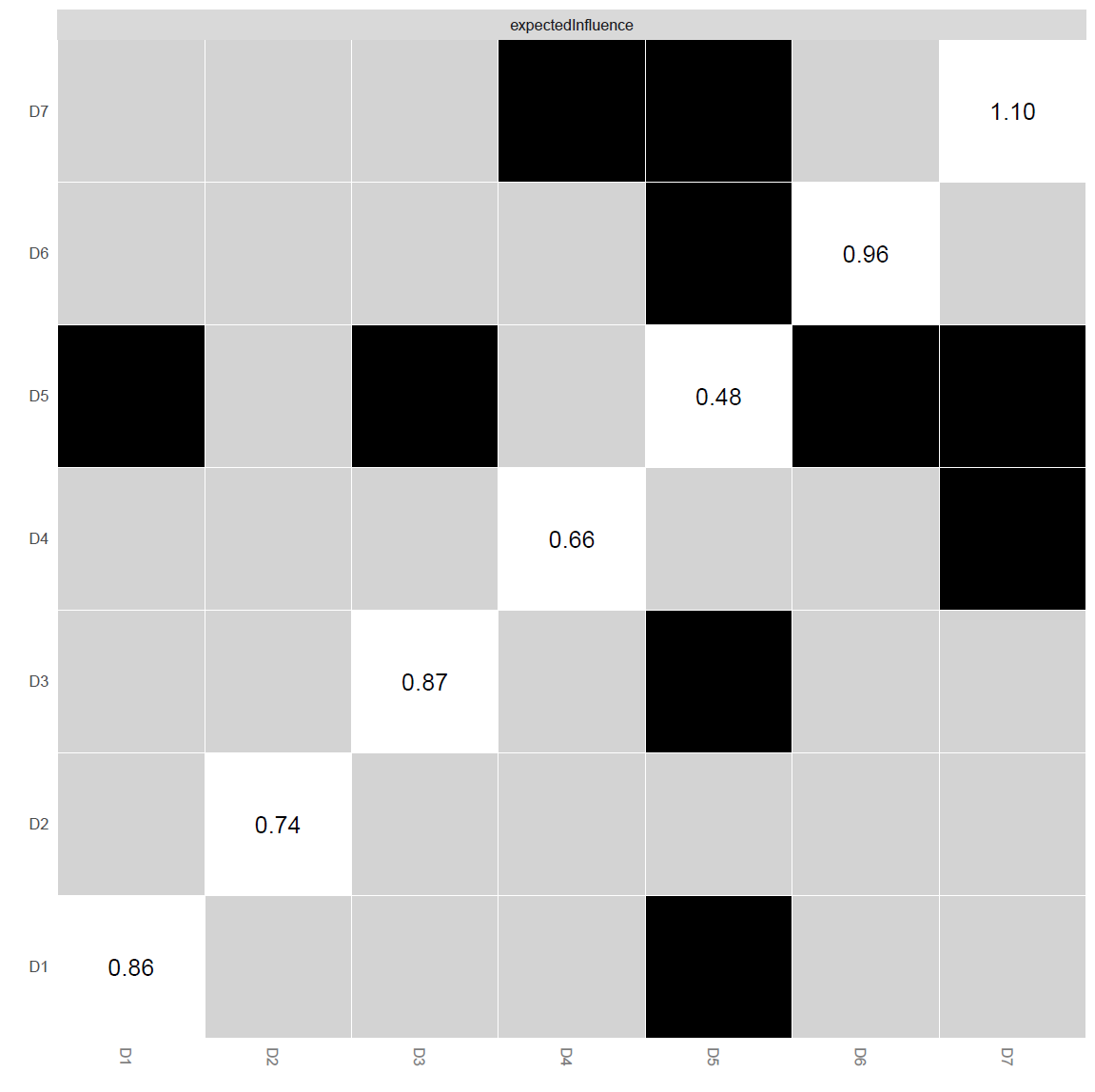


*D1: Tolerance, D2: Withdrawal, D3: Use longer than planned, D4: Unable to stop, D5: Time spent, D6: Interferes with work/life, D7: Physical/mental problems*

### Figure S4. Bootstrap difference edge weights of AAS dependence network


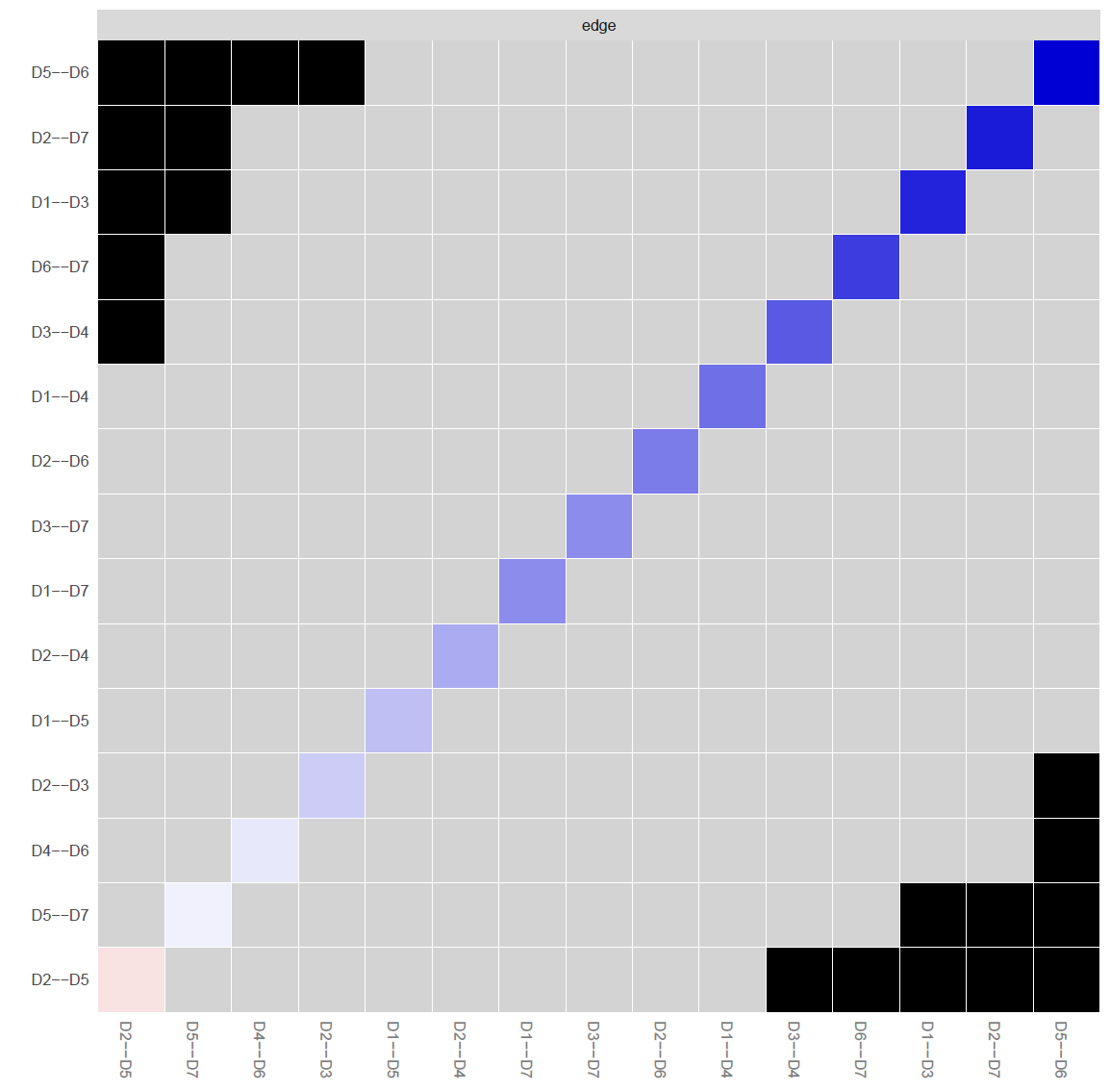


*D1: Tolerance, D2: Withdrawal, D3: Use longer than planned, D4: Unable to stop, D5: Time spent, D6: Interferes with work/life, D7: Physical/mental problems*

## MDI network: AAS users

### Figure S5. Expected influence stability of muscle dysmorphia network in AAS users


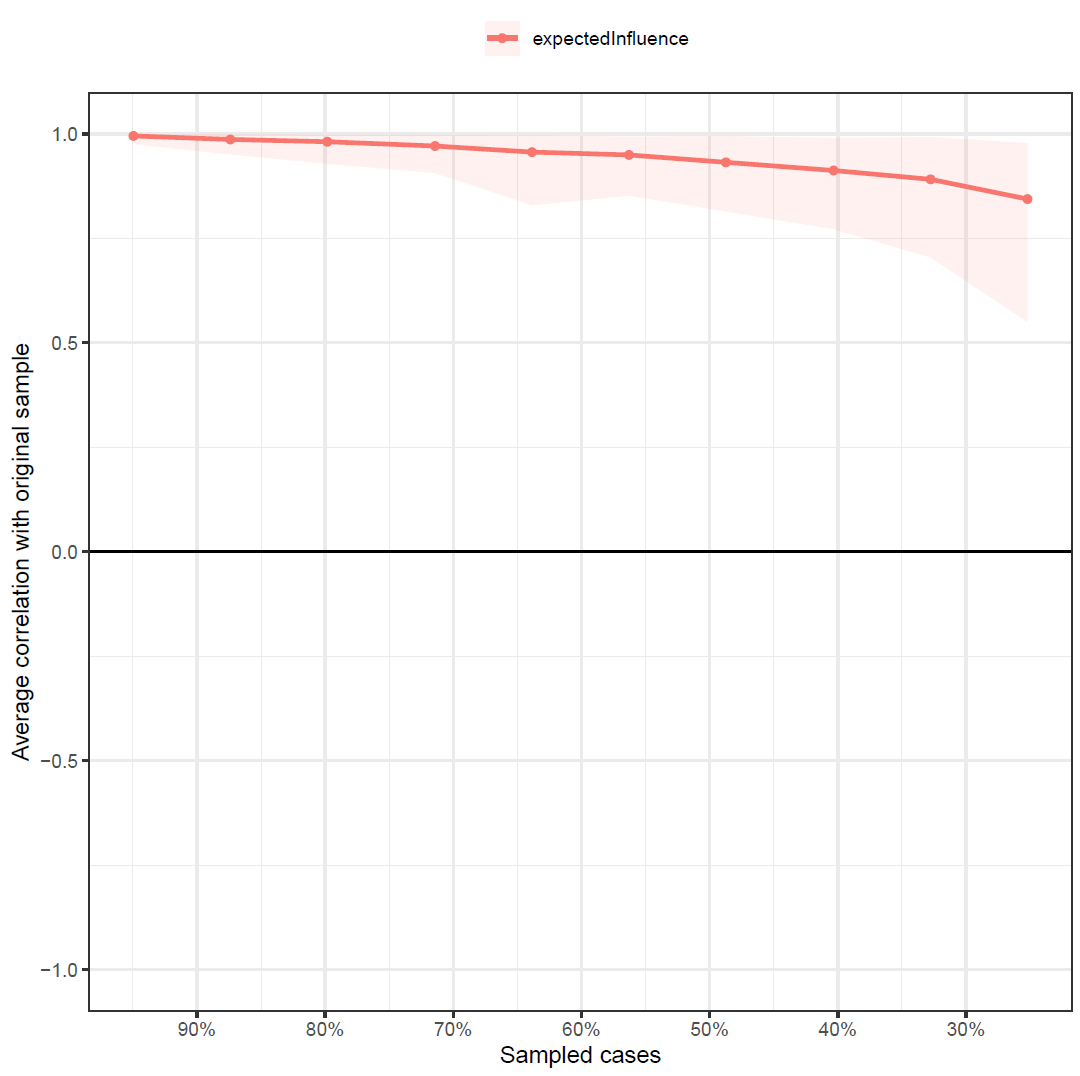


### Figure S6. Edge weight stability of muscle dysmorphia network in AAS users


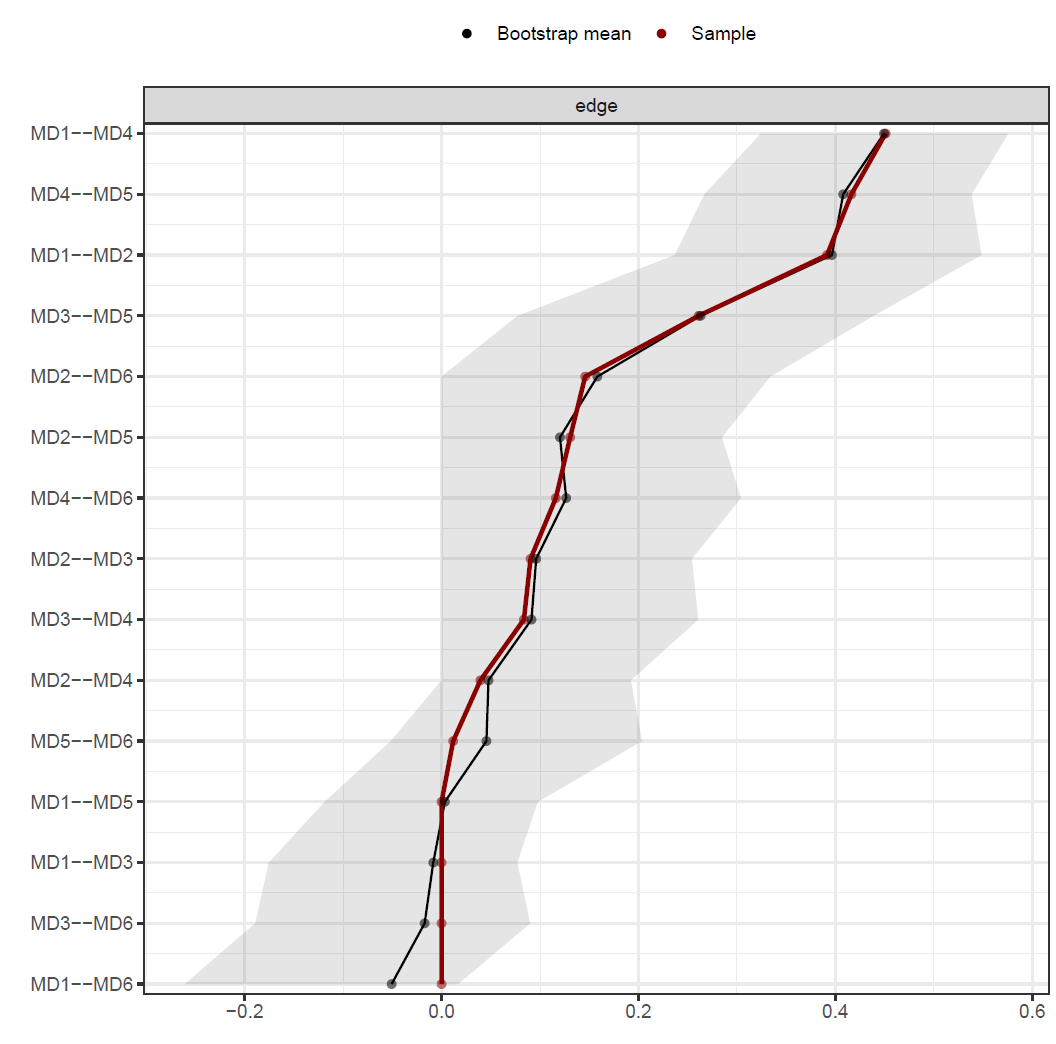


*MD1: Diet, MD2: Supplement, MD3: Physique concealment, MD4: Exercise dependence, MD5: Size/symmetry concerns, MD6: Pharmacological concerns*

### Figure S7. Bootstrap difference node expected influence of muscle dysmorphia network in AAS users


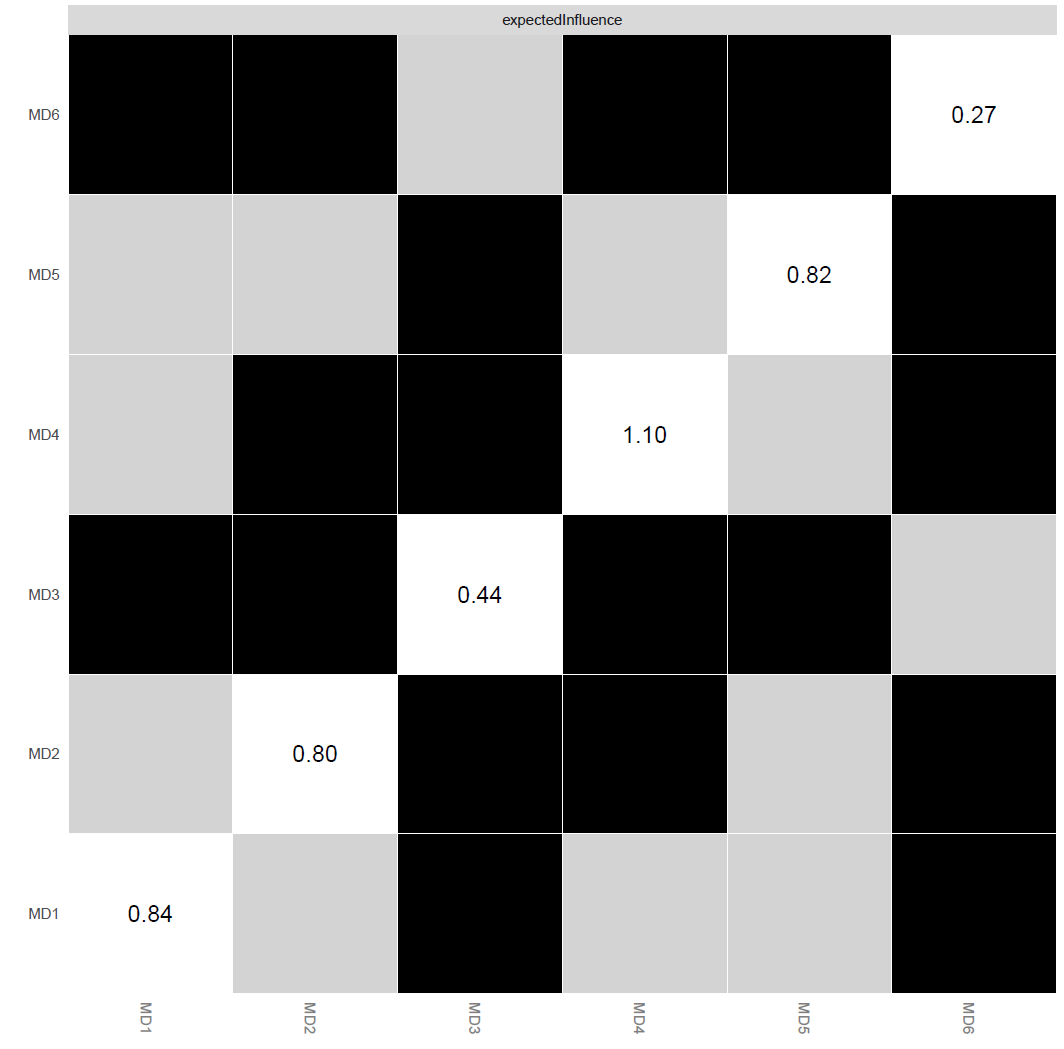


*MD1: Diet, MD2: Supplement, MD3: Physique concealment, MD4: Exercise dependence, MD5: Size/symmetry concerns, MD6: Pharmacological concerns*

### Figure S8. Bootstrap difference edge weight of muscle dysmorphia network in AAS users


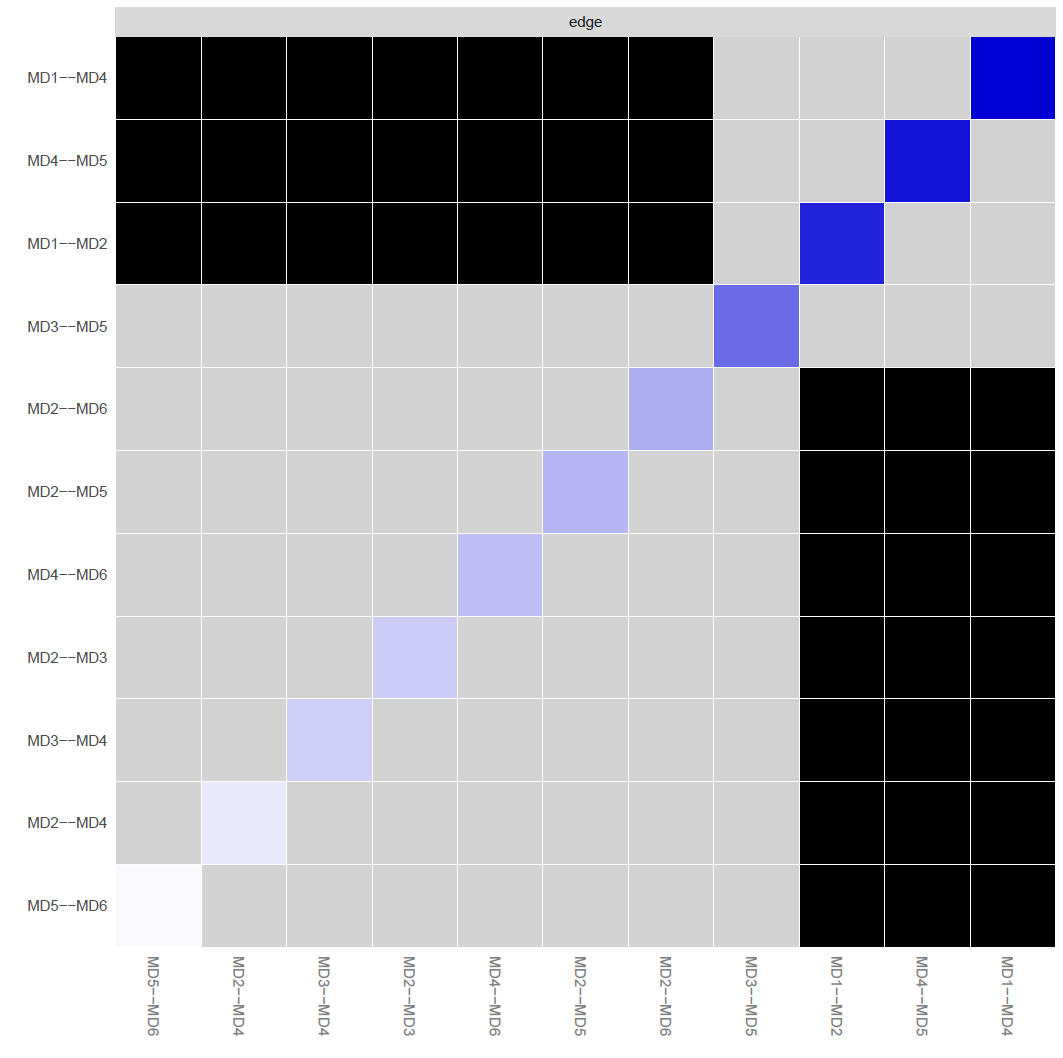


*MD1: Diet, MD2: Supplement, MD3: Physique concealment, MD4: Exercise dependence, MD5: Size/symmetry concerns, MD6: Pharmacological concerns*

## MDI network: WLC

### Figure S9. Expected influence stability of muscle dysmorphia network in WLC


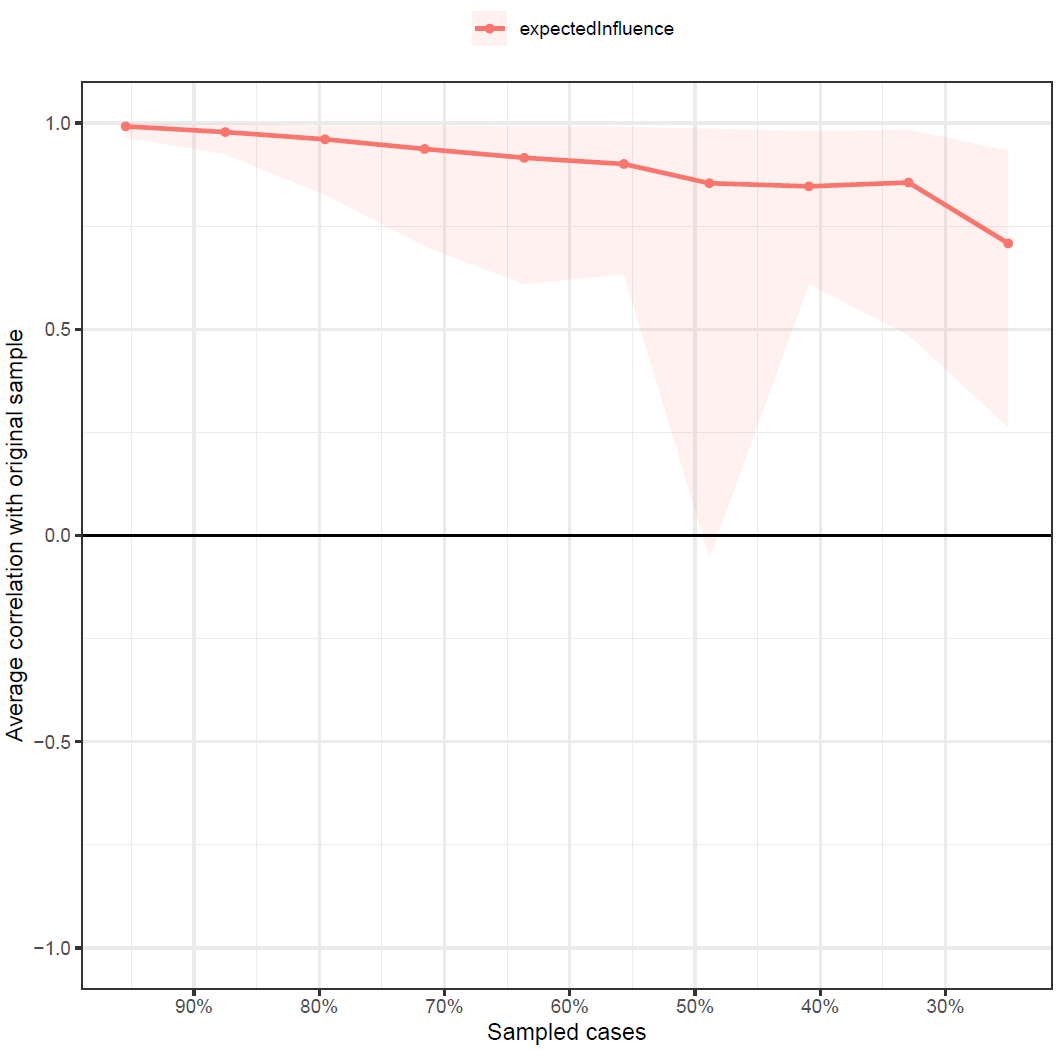


### Figure S10. Edge weight stability of muscle dysmorphia network in WLC


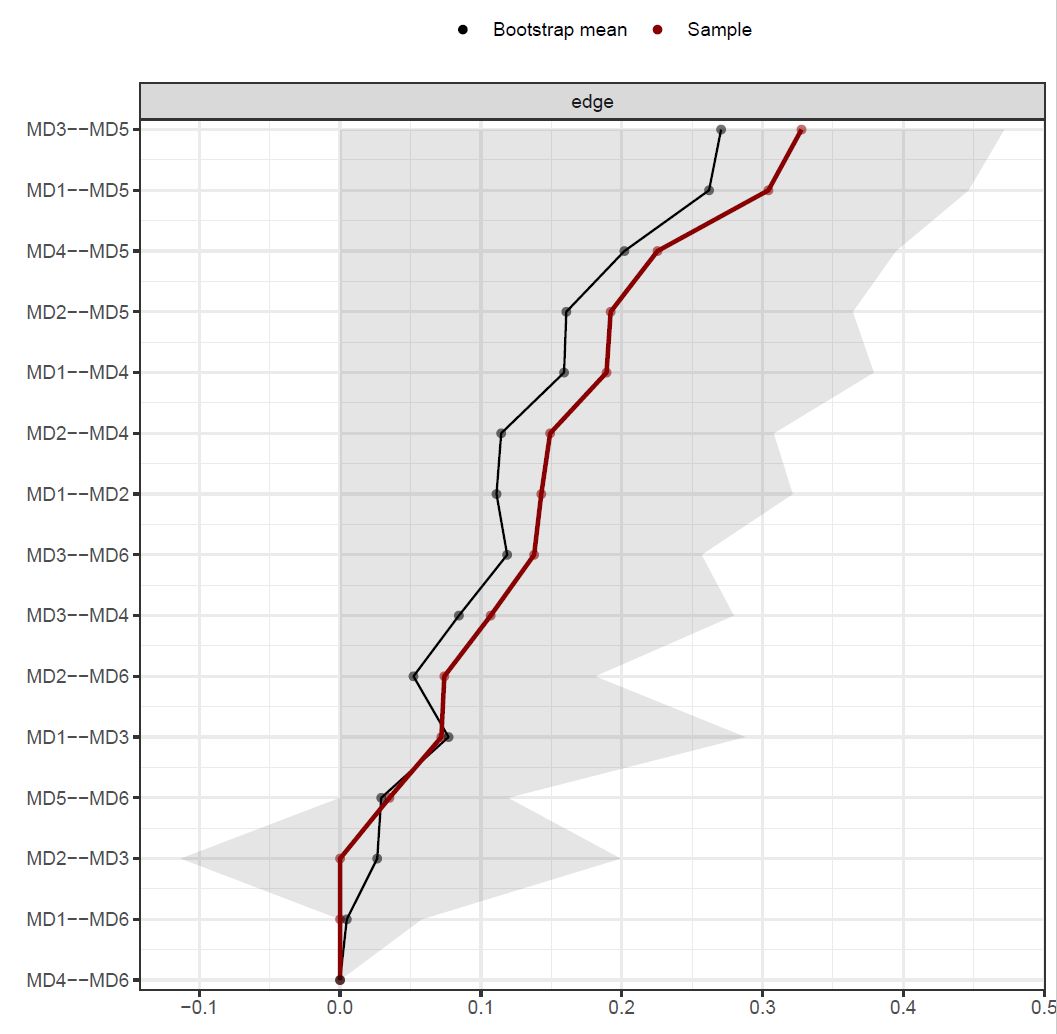


*MD1: Diet, MD2: Supplement, MD3: Physique concealment, MD4: Exercise dependence, MD5: Size/symmetry concerns, MD6: Pharmacological concerns*

### Figure S11. Bootstrap difference expected influence of muscle dysmorphia network in WLC


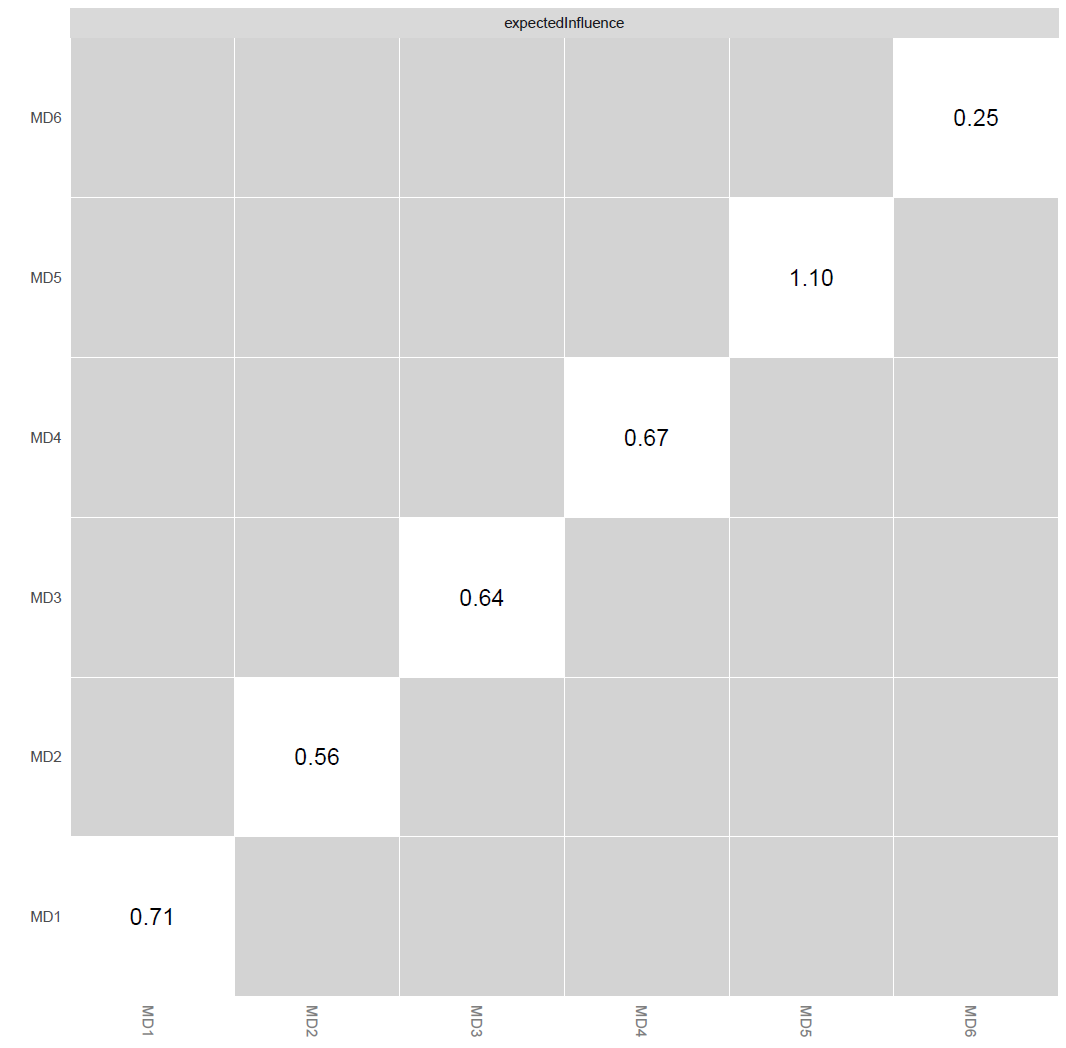


*MD1: Diet, MD2: Supplement, MD3: Physique concealment, MD4: Exercise dependence, MD5: Size/symmetry concerns, MD6: Pharmacological concerns*

### Figure S12. Bootstrap difference edge weights of muscle dysmorphia network in WLC


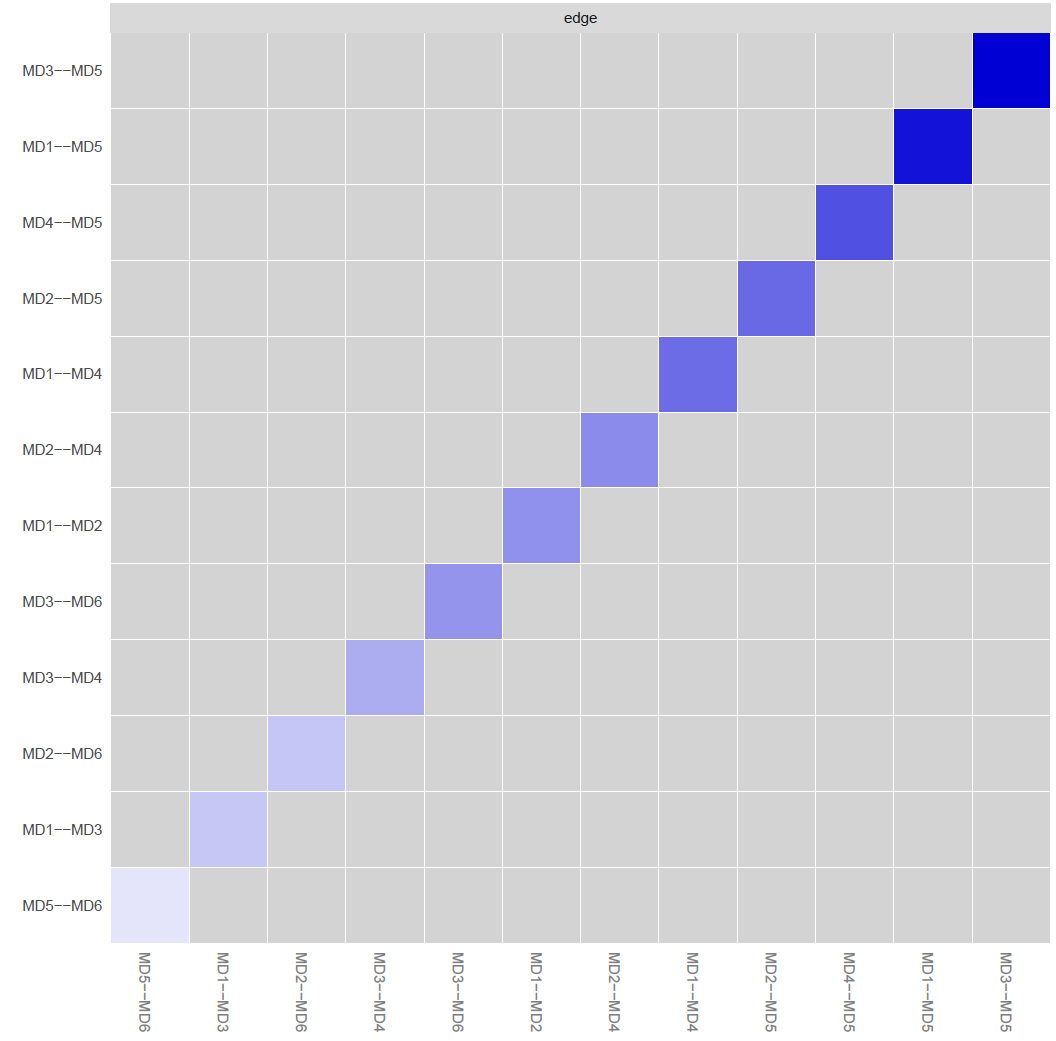


*MD1: Diet, MD2: Supplement, MD3: Physique concealment, MD4: Exercise dependence, MD5: Size/symmetry concerns, MD6: Pharmacological concerns*

## Table S1. Network Comparison Test results

|  |  |  |  |  |
| --- | --- | --- | --- | --- |

*NETWORK INVARIANCE TEST*

Test statistic M: 0.3041389

*p*= 0.157

GLOBAL STRENGTH INVARIANCE TEST

Global strength per group: 2.137931 1.956241

Test statistic S: 0.1816901

*p*= 0.203

EDGE INVARIANCE TEST

Var1 Var2 *p*

MD1 MD2 0.070

MD1 MD3 0.508

MD2 MD3 0.523

**MD1 MD4 0.035**

MD2 MD4 0.457

MD3 MD4 0.867

**MD1 MD5 0.003**

MD2 MD5 0.684

MD3 MD5 0.626

MD4 MD5 0.100

MD1 MD6 1.000

MD2 MD6 0.588

MD3 MD6 0.289

MD4 MD6 0.120

MD5 MD6 0.882

CENTRALITY INVARIANCE TEST

closeness betweenness strength expectedInfluence

MD1 0.1226 0.1444 0.3964 0.3920

MD2 0.3310 0.7926 0.1090 0.1086

MD3 0.1506 0.0612 0.2570 0.2490

**MD4 0.0032 0.0018 0.0104 0.0092**

MD5 0.6478 0.8458 0.2528 0.2534

MD6 0.6492 1.0000 0.8742 0.8712

*MD1: Diet, MD2: Supplement, MD3: Physique concealment, MD4: Exercise dependence, MD5: Size/symmetry concerns, MD6: Pharmacological concerns*

## Dependence + MDI network

### Figure S13. Expected influence stability of dependence and muscle dysmorphia network


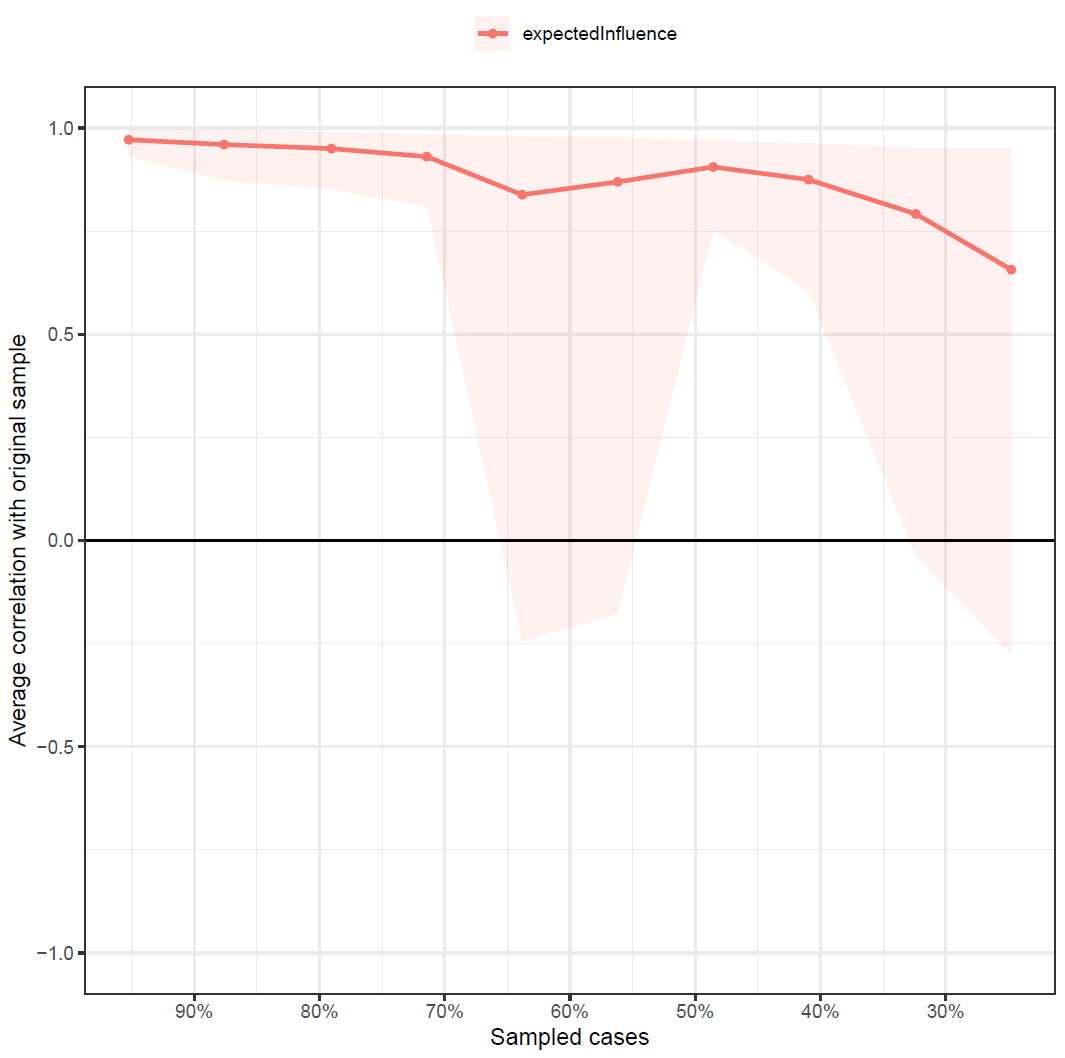


###
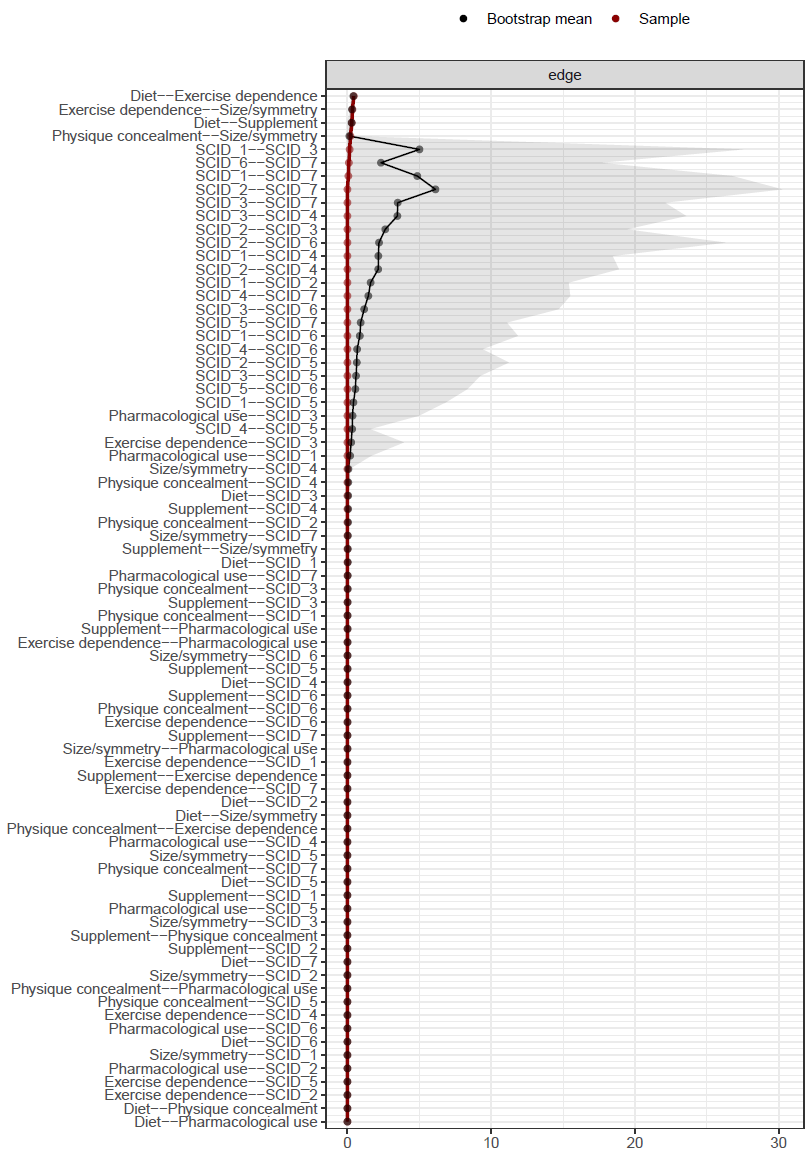
Figure S14. Edge weight stability of dependence and muscle dysmorphia network

###
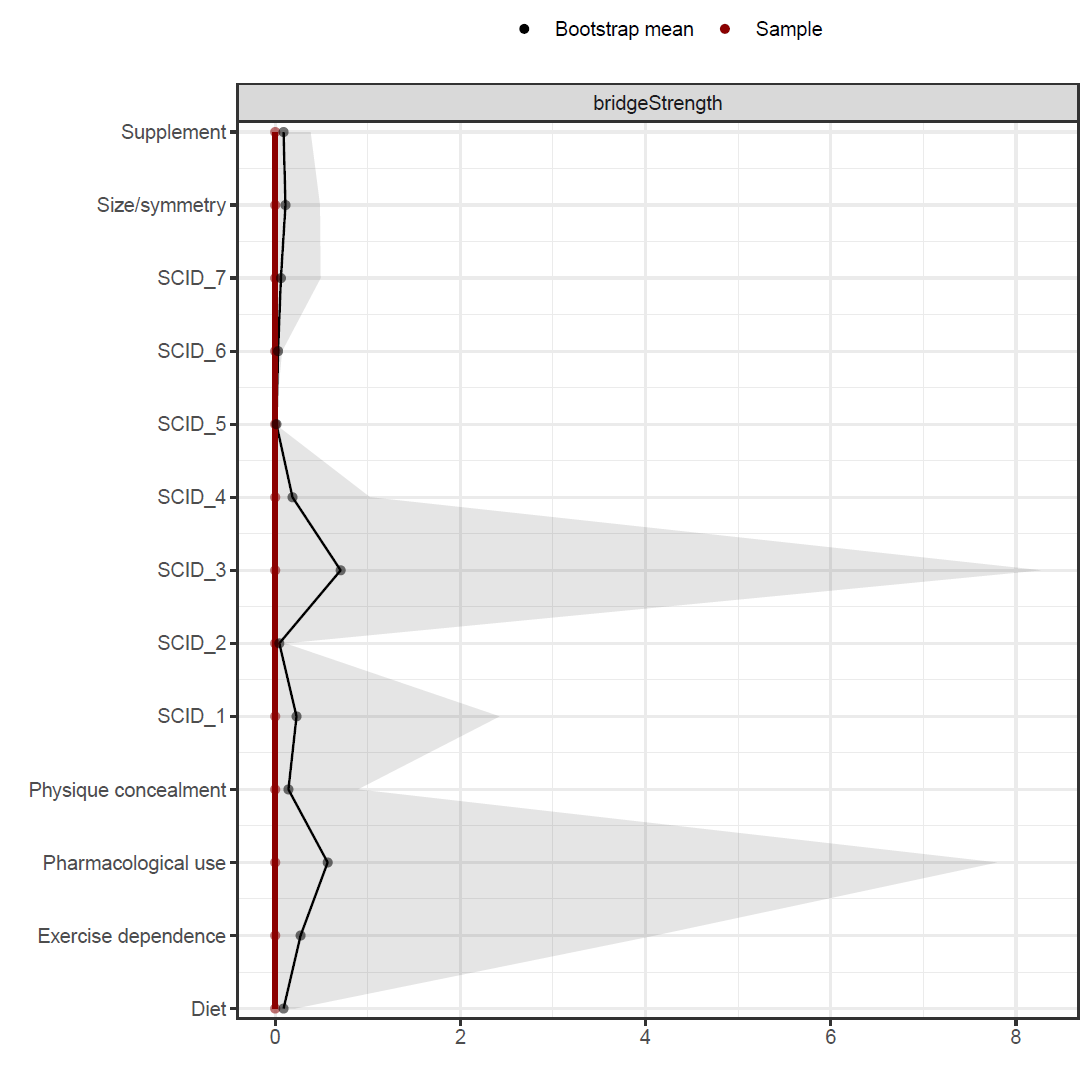
Figure S15. Bridge strength stability of dependence and muscle dysmorphia network

*SCID1: Tolerance, SCID2: Withdrawal, SCID3: Use longer than planned, SCID4: Unable to stop, SCID5: Time spent, SCID6: Interferes with work/life, SCID7: Physical/mental problems*

### Figure S16. Bootstrap difference expected influence of dependence and muscle dysmorphia network


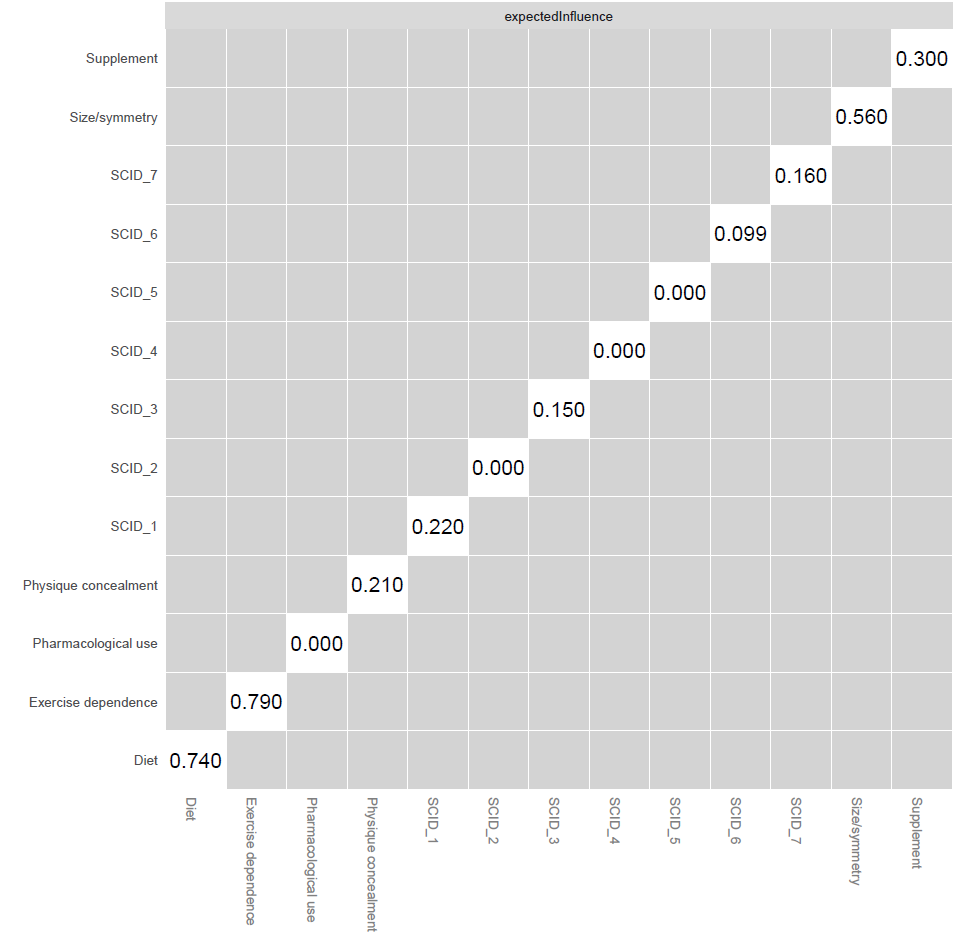


*SCID1: Tolerance, SCID2: Withdrawal, SCID3: Use longer than planned, SCID4: Unable to stop, SCID5: Time spent, SCID6: Interferes with work/life, SCID7: Physical/mental problems*

### Figure S17. Bootstrap difference edge weights of dependence and muscle dysmorphia network


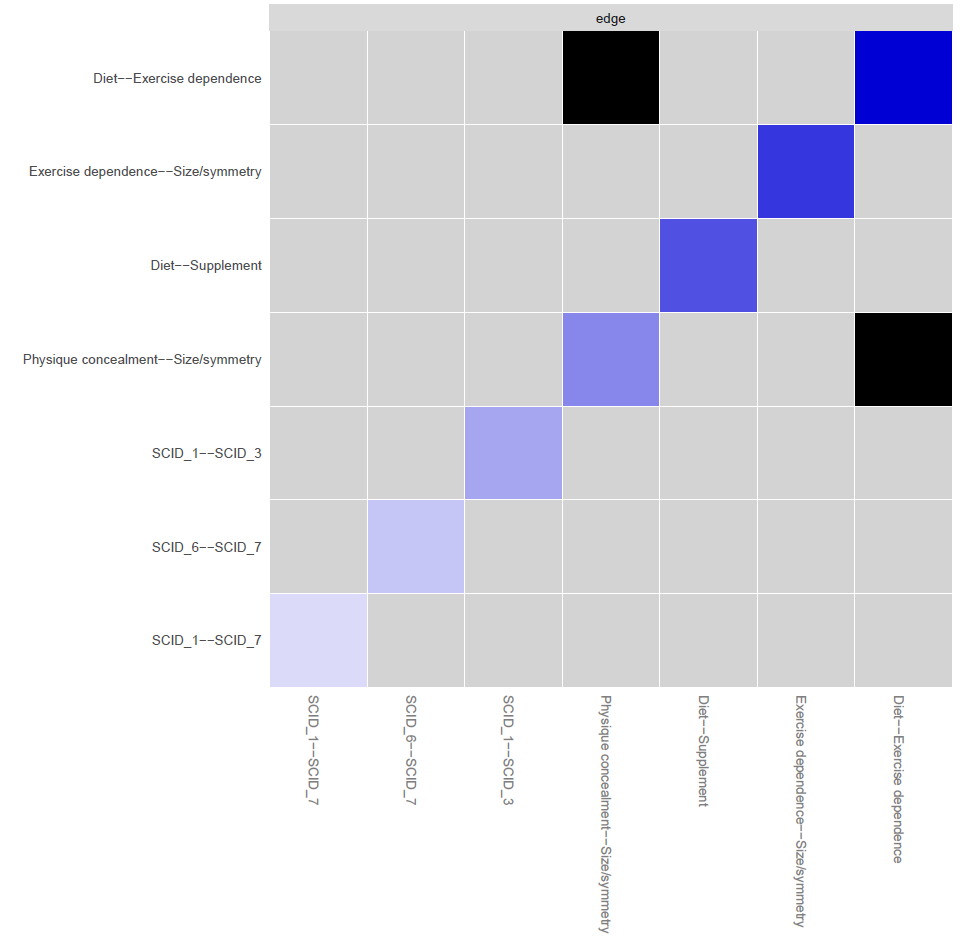
 *SCID1: Tolerance, SCID2: Withdrawal, SCID3: Use longer than planned, SCID4: Unable to stop, SCID5: Time spent, SCID6: Interferes with work/life, SCID7: Physical/mental problems*

### Figure S18. Bootstrap difference bridge strength of dependence and muscle dysmorphia network


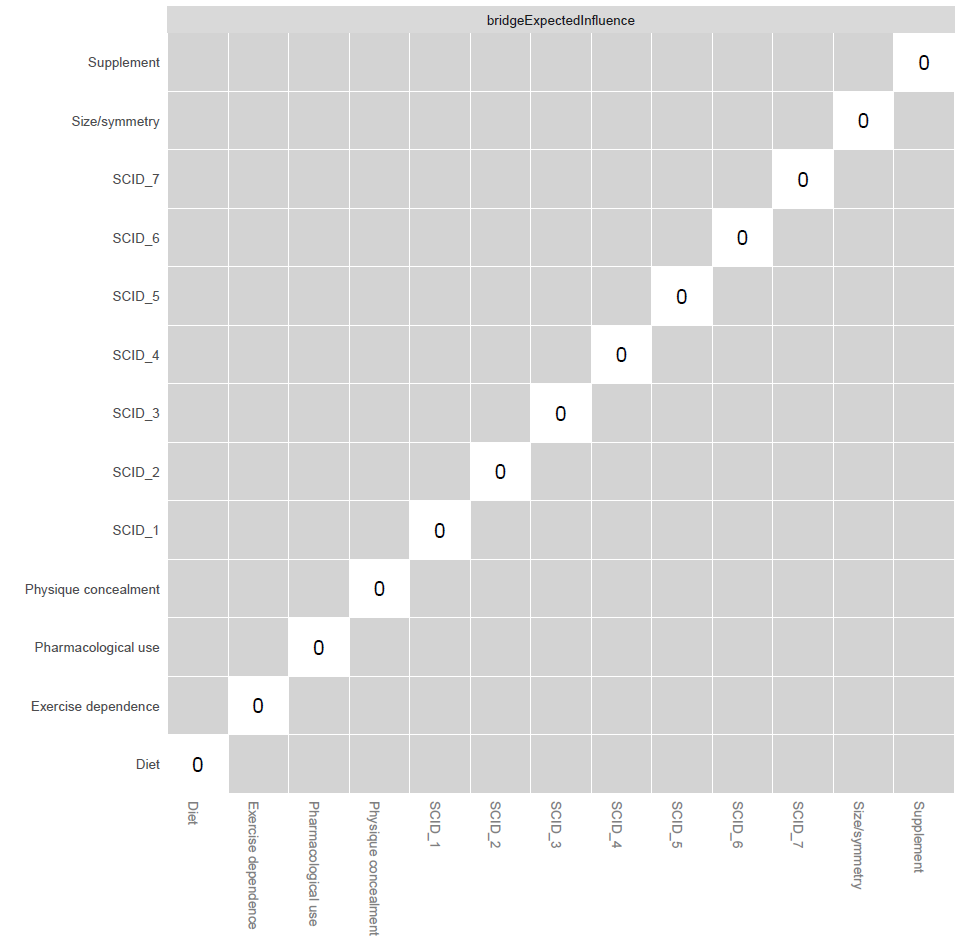


*SCID1: Tolerance, SCID2: Withdrawal, SCID3: Use longer than planned, SCID4: Unable to stop, SCID5: Time spent, SCID6: Interferes with work/life, SCID7: Physical/mental problems*
